# Fibronectin Deposition Participates in Extracellular Matrix Assembly and Vascular Morphogenesis

**DOI:** 10.1371/journal.pone.0147600

**Published:** 2016-01-26

**Authors:** Abigail Hielscher, Kim Ellis, Connie Qiu, Josh Porterfield, Sharon Gerecht

**Affiliations:** 1 Department of Chemical and Biomolecular Engineering, Johns Hopkins University, Baltimore, Maryland, 21218, United States of America; 2 Johns Hopkins Physical Sciences-Oncology Center, Johns Hopkins University, Baltimore, Maryland, 21218, United States of America; 3 Institute for NanoBioTechnology, Johns Hopkins University, Baltimore, Maryland, 21218, United States of America; 4 Department of Biomedical Sciences, Georgia Philadelphia College of Osteopathic Medicine, Suwanee, Georgia, 30024, United States of America; University of Bergen, NORWAY

## Abstract

The extracellular matrix (ECM) has been demonstrated to facilitate angiogenesis. In particular, fibronectin has been documented to activate endothelial cells, resulting in their transition from a quiescent state to an active state in which the cells exhibit enhanced migration and proliferation. The goal of this study is to examine the role of polymerized fibronectin during vascular tubulogenesis using a 3 dimensional (3D) cell-derived de-cellularized matrix. A fibronectin-rich 3D de-cellularized ECM was used as a scaffold to study vascular morphogenesis of endothelial cells (ECs). Confocal analyses of several matrix proteins reveal high intra- and extra-cellular deposition of fibronectin in formed vascular structures. Using a small peptide inhibitor of fibronectin polymerization, we demonstrate that inhibition of fibronectin fibrillogenesis in ECs cultured atop de-cellularized ECM resulted in decreased vascular morphogenesis. Further, immunofluorescence and ultrastructural analyses reveal decreased expression of stromal matrix proteins in the absence of polymerized fibronectin with high co-localization of matrix proteins found in association with polymerized fibronectin. Evaluating vascular kinetics, live cell imaging showed that migration, migration velocity, and mean square displacement, are disrupted in structures grown in the absence of polymerized fibronectin. Additionally, vascular organization failed to occur in the absence of a polymerized fibronectin matrix. Consistent with these observations, we tested vascular morphogenesis following the disruption of EC adhesion to polymerized fibronectin, demonstrating that block of integrins α_5_β_1_ and α_v_β_3,_ abrogated vascular morphogenesis. Overall, fibronectin deposition in a 3D cell-derived de-cellularized ECM appears to be imperative for matrix assembly and vascular morphogenesis.

## Introduction

Angiogenesis is a hallmark of tumor formation, supplying the tumor mass with the oxygen and nutrients necessary for meeting its voracious metabolic demands. In addition, angiogenesis is a requisite for the successful transplantation of tissue engineered scaffolds, where the delivery of oxygen and nutrients is imperative for cell growth and thus restoration of the damaged tissue. In this manner, a better understanding of the mechanisms regulating angiogenesis is necessary for targeted disruption of angiogenesis in tumors and enhancement of angiogenesis in transplanted tissues. While numerous factors participate in angiogenesis, recent efforts have focused on the role of the ECM in pathological and non-pathological angiogenesis. Of particular interest is the ECM protein fibronectin.

Fibronectin is a large glycoprotein which plays an essential role in development, wound healing, tumorigenesis and angiogenesis. With regard to angiogenesis, the absence of fibronectin in mice was reported to be lethal [1, 2]. In these studies, mice lacking fibronectin presented with deformed embryonic vessels and die during embryogenesis as a result of severe cardiovascular defects [1, 2], supporting a crucial role for fibronectin in vascular morphogenesis. More recent studies have specifically shown that fibronectin participates in angiogenesis via its role in promoting EC activation, survival, migration, proliferation and elongation [3–6], crucial steps in the angiogenic cascade. Work in our lab has shown that patterned fibronectin surfaces guided the attachment and elongation of endothelial progenitor cells[7]. Others have demonstrated that a 3D fibrin-rich matrix promoted vascular morphogenesis of ECs, with EC-derived fibronectin reported to play a critical role in regulation of this process [8]. Fibronectin has also been reported to play a role in vascular remodeling. Specifically, Chiang et al [9] showed that application of a pluronic gel complexed with a peptide inhibitor of fibronectin polymerization reduced vascular wall thickening in mice which had undergone surgical ligation of the left carotid artery [9]. *In-vivo* studies have found that teratocarcinomas derived from embryonic stem cells null for α_5_ integrin, a receptor promoting cellular attachment to fibronectin, expressed significantly fewer vascular structures in comparison to α_5_ integrin-expressing cells [10], further supporting a role for fibronectin in angiogenesis These results highlight the important role of fibronectin in directing vascular cell behaviors and angiogenic activities.

Fibronectin is produced by several cell types including fibroblasts [11–13] and is a component of the ECM milieu of several organs. Here, we describe for the first time, the use of a completely biological 3D de-cellularized ECM [11, 14] rich in fibronectin for analyses of vascular morphogenesis and matrix assembly. Using this 3D matrix as a culture platform, we tested the hypothesis that both fibronectin polymerization in the matrix and fibronectin deposition by ECs participate in vascular morphogenesis of ECs on the de-cellularized ECM. We demonstrate that matrix fibronectin was indispensable for vascular morphogenesis and matrix assembly as interference of EC attachment to fibronectin abrogated vascular morphogenesis and loss of a polymerized fibronectin matrix in the de-cellularized ECM not only prevented vascular organization, but markedly reduced deposition of other matrix proteins. Furthermore, our data point to a role for a polymerized fibronectin matrix in EC migration where its presence in the matrix was found to decrease EC migration patterns during vascular morphogenesis. Utilizing a completely biological system, our results support a novel role for fibronectin in vascular morphogenesis and matrix assembly *in-vitro*. Combined, these results have important implications for understanding the mechanisms whereby fibronectin may direct vascular morphogenesis and matrix assembly, results which are applicable to the fields of both regenerative medicine and cancer biology.

## Materials and Methods

### Cell Lines and Culture

The MDA231 (MDA) breast cancer cell line was a gift from the Physical Sciences-Oncology Bioresource Core Facility (PBCF, ATCC; Manassas, VA) and were originally obtained through the laboratory of Dr. Thea Tlsty (University of California San Francisco, San Francisco, CA). The human neonatal foreskin fibroblast (NuFF) cell line was obtained from Global Stem (Rockville, MD; #GSC-3002) at passage 9. Human Umbilical Vein Endothelial Cells (HUVECs) were obtained from Promocell (Heidelberg, Germany). MDA231 cells were cultured in DMEM (Life Technologies, Grand Island, NY) supplemented with 10% vol/vol FBS (Atlanta Biologicals). NuFF cell were cultured in DMEM supplemented with 10% vol/vol heat inactivated FBS (Life Technologies) and HUVECs were cultured in EGM media (Promocell) supplemented with 2% FBS (Promocell). Media was exchanged every 2–3 days and cells were passaged after reaching 80–90% confluency using 0.25% trypsin EDTA (Sigma, Allentown, PA) or 0.05% typsin EDTA (for HUVECs) (Sigma). All cell lines were maintained at 37°C in a humidified atmosphere containing 5% CO_2_.

### Antibodies

Primary antibodies include: rabbit anti-human fibronectin, mouse anti-human collagen I, mouse anti-human collagen IV, rabbit anti-human laminin, mouse anti-human tenasin-C, mouse anti-human CD31 and phalloidin 488 or 546. Secondary antibodies include: goat anti-mouse Cy3, goat anti-mouse FITC and goat anti-rabbit alexa fluor 488. Refer to S1 Table for information on all antibodies including dilutions and suppliers. Blocking antibodies utilized in the study include: β_1_ (clone Mab13, BD Biosciences; San Jose, CA), α_5_ (clone IIa1, BD Biosciences), and α_v_β_3_ (Clone LM609, Millipore, Billerica, MA). IgG isotype control antibodies were mouse IgG1 κ (clone MOPC-21, BD Biosciences) and rat IgG2a κ (Clone R35-95, BD Biosciences).

### Peptides

The fibronectin inhibitor peptide pUR4B and control III-11C peptide (kindly provided by Dr. Jane Sottile, Rochester University) were obtained from cloning into Escherichia Coli and isolated as previously described [9]. Endotoxin was removed from peptides and residual levels measured as previously described [9].

### GFP transduction

A lentiviral vector was used for GFP transduction of ECs. Plasmids pMD2.G, CMV 11904, and iDUET 11426 were kindly provided by Dr. Linzhao Cheng (Johns Hopkins University). Methods used for transformation and transfection were based off of methods described by [15]. In brief, 10pg to 100ng of plasmids were transformed into OneShot Stlb3 cells (Life Technologies) according to the manufacturer’s instructions. A volume of 25μl of the transformed cells was plated on LB agar plates containing 100μg/ml of ampicillin and allowed to grow overnight at 37°C. The next day, colonies were selected and grown overnight in 5ml of LB medium containing 100μg/ml of ampicillin in a shaker incubator set at 37°C. The Qiagen Spin Miniprep Kit was used to isolate bacterial DNA (Qiagen, Germantown, MD). For transfection, Hek293t3 cells (ATCC, Manassas, VA) were plated at 500,000 cells in a 6 well and allowed to attach overnight. The next day, the media was exchanged to 1% serum and HEK293 cells were transfected with the following concentrations of plasmids: 2μg of CMV, 0.5μg of PMD.G, and 1μg of iDUET in lipofectamine (Life Technologies). Methods were based off of manufacturer’s instructions. After two days of transfection, the supernatant was collected and the virus concentrated using an Amicon Ultracentrifugal filter (Millipore) and subsequently stored at -80°C. For transduction, HUVECs (at passage 1) were plated in a 6-well plate at 100,000 cells/well. The virus was added at a ratio of 1:100 (vol/vol) virus to HUVEC medium and incubated at 37°C, 5% CO_2_ in a humidified atmosphere for 24 hours. The media was changed after the 24-hour viral incubation. The cells were gently scraped from the wells, spun down at 800 rpm for 3 minutes, and suspended in 300μl of 1X PBS. This suspension was filtered using a 40-um mesh strainer (BD Biosciences) and transferred in 5 ml FACs tubes for sorting. HUVECs expressing a high level of GFP were sorted and isolated using FACS Aria II Sorter with a 488nm laser at the Ross Research Flow Cytometry Core Facility (Johns Hopkins University School of Medicine, Baltimore, MD).

### Co-cultures

NuFF (passages 18–28) were co-cultured with MDA breast cancer cells for establishment of cell-derived ECM. Both cell lines were seeded on the same day in a 1:1 ratio in 4 well Nunc LabTek II Chamberslides (Sigma) using methods previously described [11, 14]. In brief, co-cultures were maintained in one-half NuFF media and one-half MDA media with a final concentration of 10% fetal bovine serum (FBS). In some instances, chamberslides were coated with 5ug/well human fibronectin (Sigma) for 1 hour at 37°C, 5% CO_2_ in a humidified atmosphere prior to cell seeding. Media was exchanged every 2–3 days and co-cultures were maintained for 7–9 days before de-cellularization.

### Matrix de-cellularization and seeding of HUVECs

Isolation of cell-derived ECM and subsequent seeding of HUVECs for vascular morphogenesis were based off of methods described in prior published reports [11, 14]. HUVECs were used at passages 4–5 for all analyses of vascular morphogenesis on de-cellularized ECM and were cultured in EGM media containing 2% serum.

### Inhibition of fibronectin polymerization

NuFF/MDA co-cultures were incubated in 250nM of pUR4B or control III-11C peptides. Fresh inhibitor and control peptides were added every day along the culture period. For analysis of the contribution of HUVEC-derived fibronectin to vascular morphogenesis, HUVECs were seeded on intact, de-cellularized ECM in the presence of 500nM of pUR4B or control III-11C peptides. NuFF/MDA co-cultures and vascular structures on de-cellularized ECM were evaluated for fibronectin expression using qRT-PCR and western blot as described below.

### Inhibition of collagen synthesis

NuFF cells were seeded at 45,000 cells/well in Nunc LabTek II Chamberslides and were incubated in media with or without 100nM of halofuginone (Sigma) for 24 hours. Serum concentrations were adjusted to 2.5% serum or 5% serum. Cells were fixed and evaluated using immunofluorescence (described below) for collagen I, fibronectin and tenascin-C matrix expression.

### Immunofluorescence staining and imaging of ECM and vascular structures

De-cellularized NuFF/MDA ECM and vascular structures were fixed and prepared as previously described [11, 14]. The de-cellularized ECM was stained with fibronectin and collagen I or IV, laminin or tenasin-C. Vascular structures were stained with either CD31, phalloidin or one of each of the ECM proteins: fibronectin, collagens I or IV, laminin or tenasin-C. ECM and vascular structures were incubated in secondary antibodies Refer to S1 Table for information on all antibodies used including dilutions and suppliers. Cellular nuclei were visualized using DAPI at a dilution of 1:1,000 (Life Technologies). All immunolabeled samples were mounted and imaged as previously described [11, 14].

### Confocal and time-lapse imaging

Live cell time-lapse images were taken from hour 7 to hour 12 following GFP-HUVEC seeding on ECM using a Zeiss LSM 510 Meta Confocor 3 (Carl Zeiss; Integrated Imaging Center; Johns Hopkins University). Fluorescent z-stack images of step size 2–3 μM were taken with a 20x objective (Zeiss) every 15 minutes for 5 hours. An argon (488nm) laser was used to obtain the fluorescent images. Multi-Time 4.0.31 (Zeiss) was used to set up the time-lapse and Zen (Zeiss) was used to set up the image configurations. During time-lapse imaging, the cells were cultured and incubated using previously mentioned conditions. Fixed, immunolabeled vascular structures were evaluated for the presence of lumens and evidence of vascular organization. A step size of 0.70μM was used and images were acquired at 40 and 63x magnification.

### Scanning electron microscopy

Scanning electron microscopy was performed as previously described [11, 14]. Three coverslips each containing de-cellularized ECM deposited from pUR4B and control III-11C treated NuFF/MDA co-cultures were evaluated for the extent of ECM expression.

### Real-time quantitative RT-PCR

Two-step RT-PCR was performed on cDNA from NuFF/MDA co-cultures or vascular structures from HUVECs grown for 24 hours on de-cellularized NuFF/MDA ECM. NuFF/MDA co-cultures were treated daily with 250nM of pUR4B or control III-11C or were left untreated for a period of 9 days. HUVECs were treated with 500nM of pUR4B or control III-11C for a period of 24 hours or were left untreated. Total RNA was extracted, quantified, and reverse transcribed into cDNA as previously described [11, 14]. The TaqMan Universal PCR Master Mix and Gene Expression Assay (Applied Biosystems, Foster City, CA) were used for analyses of fibronectin and the following matrix metalloproteases (MMPs): MMP1, MMP2, MMP9 and MT1-MMP (Applied Biosystems) according to the manufacturer’s instructions. The TaqMan PCR step was performed with an Applied Biosystems StepOne Real-Time PCR System (Applied Biosystems), following the manufacturer’s instructions. The relative expression of fibronectin and MMPs was normalized to the amount of β-actin or GAPDH (Applied Biosystems) in the same cDNA through use of the standard curve method described by the manufacturer. For each primer set, the comparative computerized tomography method (Applied Biosystems) was used to calculate amplification differences between untreated, control III-11C and pUR4B treated samples. The values for experiments were averaged and graphed with standard deviations.

### Enzyme Zymography

HUVECs at passage 5 were grown on de-cellularized ECM from NuFF/MDA co-cultures. After 12 hours in EGM media supplemented with 2% serum, the media was exchanged to serum free EGM media for the remaining 12 hours of vascular assembly. Supernatant was collected, stored long term at -80°C and thawed on ice during experimental analyses. The supernatant was diluted 1:1 with Laemmli buffer without addition of reducing agents. A volume of 40μl was loaded on a 12% casein gel (Life Technologies) for MMP1 or a 10% gelatin gel (Life Technologies) for MMPs 2 and 9. The gels were run at 150V for 1.5 hours in SDS running buffer, followed by a series of four 15 minute washes in 1X Renaturation buffer (Life Technologies). The gels were transferred to 1X Denaturation Buffer (Life Technologies) for 15 minutes with gentle shaking and then placed at 37°C for incubation overnight. The following day, the gels were fixed in a solution containing 50% methanol and 10% acetic acid for 30 minutes and stained in 0.02% Coomassie (Sigma) in 50% methanol and 10% acetic acid for 2 hours. The gels were de-stained in 20% methanol, 10% acetic acid solution for 1–2 hours and transferred to deionized H_2_O and imaged using ChemiDoc^TM^ XRS+ System (Biorad, Hercules, CA). Images were acquired using Biorad Quantity One^TM^ software. Differences between MMPs 2 and 9 on gelatin zymograms were distinguished based on known molecular weights where ~90 kDa corresponds to proMMP9 and ~72 kDa and ~62 kDa correspond to the pro and active forms, respectively, for MMP2 [16, 17].

### Western Blot

NuFF/MDA co-cultures or vascular structures from HUVECs grown on de-cellularized co-culture ECM were utilized for western blot analyses. NuFF/MDA co-cultures were treated daily with 250nM of pUR4B or control III-11C or were left untreated for a period of 9 days. HUVECs were treated with 500nM of pUR4B or control III-11C or were left untreated for a period of 24 hours. Cells were lysed and protein was quantified as previously described [11, 14]. A concentration of 20–30μg of protein was loaded per well into a 4–20% SDS PAGE gel (BioRad) and run under reducing conditions. Proteins were transferred to nitrocellulose membranes (Biorad), blocked for 1 hour in 3% non-fat milk (Biorad), and incubated overnight at 4°C/constant shaking with the following antibodies: anti-MT1-MMP, anti-fibronectin and GAPDH. Membranes were washed, incubated in secondary antibodies and imaged as previously described [11, 14]. Refer to S1 Table for dilutions and suppliers for all antibodies.

### Cell proliferation

NuFF/MDA co-cultures were treated with 250nM of pUR4B or control III-11C or were left untreated for 48 hours. After 48 hours, cell proliferation was assessed using the XTT assay (Sigma) according to the manufacture’s specifications.

### ECM co-localization with fibronectin

The percent ECM co-localized with fibronectin was analyzed from 40x magnification images acquired from an Olympus BX60 microscope. Images of ECM co-localization were taken from 3–4 non-overlapping images from a total of 2–3 wells. The percent overlap for each ECM protein with fibronectin was obtained using the Co-localization tool in Metamorph version 6.1 (Universal Imaging Co., Downingtown, PA), a plugin which provides quantitative information regarding the overlap of two fluorescent signals in an image pair. To analyze co-localization, the images were thresholded so the positively stained regions were highlighted. Thresholded images were compared to the original image to ensure accuracy of thresholding. The area of overlap in the entire image pair was then analyzed. The percentage of overlap in one image versus the other (e.g. collagen overlap with fibronectin) was recorded and expressed as the percent co-localization.

### Blocking integrins α_5_β_1_ and α_v_β_3_ on HUVECs

To inhibit vascular cell interaction with fibronectin, HUVECs were seeded in the presence of 20μg/ml each of the following blocking integrins: α_5_, β_1_, and α_v_β_3_ [18]. To address the contribution of α_v_β_3_ to vascular cell interaction with fibronectin, HUVECs were seeded with 20 μg/ml of α_v_β_3_ with and without 20 μg/ml each of α_5_ and β_1_ [18]. IgG isotype control antibodies were utilized at 20μg/ml. Vascular structures were maintained for 24 hours in culture, prior to fixation and staining as described above.

### Vascular quantification

Vascular structures were quantified from non-overlapping high magnification phalloidin stained images using the threshold function in ImageJ. Using this tool, images were first converted to a 16 bit gray scale image, were automatically threshoIded, and analyzed for the percent area covered by capillary-like structures (CLS) [14]. To illustrate the differences in pixel intensities of luminal CLS as opposed to planar sheets of ECs, we have calculated the average pixel intensities of regions with and without CLS. This analysis was conducted on 3 non-overlapping regions in each of 11 to 12 images from control III-11C and pUR4B treated ECs, respectively. To confirm that the brighter structures were indeed CLS, we used confocal imaging to generate z stacks. Analyzing z stacks of at least 15 confocal images, we documented the presence of lumens in these brighter structures and the lack of lumens in the dimmer sheets of planar ECs. This approach proved to be sufficient to determine the presence of luminal CLS and served for the purpose of this study [11, 14]. For studies on the effect of Fn inhibition during vascular morphogenesis, eight to twelve non-overlapping images were taken from each well in two wells in a total of three slides. For studies on the effect of anti-integrins on vascular morphogenesis, 9–12 non-overlapping images from each condition were evaluated.

### Particle tracking

GFP-HUVEC cell migration was tracked using the Manual Tracking plugin (Fabrice P. Cordières, Institut Curie, France) in ImageJ (NIH). The x/y calibration was set to 0.877 μm/pixel. The center of the cell was used as a reference point for each cell in each frame. Cells that entered or exited the field of view during the 5-hour tracking time interval were excluded. Cells that underwent apoptosis or proliferation were also disregarded during the tracking process. Between 60 and 100 cells were tracked per condition per experiment. Each experiment was repeated three times and each condition was in duplicate. The time interval was 15 minutes for a total of 5 hours.

### Cell migration parameters

The cell migration parameters were calculated using a custom Matlab (The Mathworks, Natick, MA) code. The XY coordinates and the distance of each particle per frame retrieved by Manual Tracking were imported into Matlab. The migration parameters calculated were total distance traveled (of 5 hours), velocity between each frame, average velocity (of 5 hours), total displacement (of 5 hours), and mean square displacement. The total distance traveled per cell was calculated by summing the interval distances,

$$D_{total}=\sum_{i=1}^{N}D_{i}$$

where D_total_ represents the total distance traveled, N is the number of distances, i represents each individual frame, and D_i_ is the distance traveled for each frame.

The velocity was calculated by dividing the interval distances by the interval time of 15 minutes,

$$V_{i}=\frac{D_{i}}{△t}$$

where V_i_ is the interval velocity, and Δt represents the time interval (15 minutes). The following equation was used to calculate the average velocity,

$$V_{avg}=\frac{1}{N}\sum_{i=1}^{N}V_{i}$$

where V_avg_ represents the average velocity and N represents the number of total velocities. The total displacement was calculated using the following equation,

$$d_{net}=\sqrt{{\left(x_{f}-x_{i}\right)}^{2}+{\left(y_{f}-y_{i}\right)}^{2}}$$

where d_net_ represents the net displacement of a cell, x_f_ is the final x position, x_i_ is the initial x position, y_f_ is the final y position, and y_i_ is the initial y position. Mean square displacement is a parameter often used to characterize the motility of cells along with the previously mentioned parameters. It was calculated using the following equation,

$$MSD=\langle {\left[r(t+\tau )-r(t)\right]}^{2}\rangle$$

where MSD presents the mean square displacement, r(t) is the position of the particle at time t, τ is the lag time between the two positions.

### Statistical Analyses

Statistical analysis was performed using GraphPad Prism 4.02 (GraphPad Software Inc., La Jolla, CA). GraphPad Prism 4.02 was used to perform ttests, One Way ANOVA with Turkey’s posttest, and Two Way ANOVA with Bonferroni’s posttest. Significance levels were set at *p≤0.05, **p≤0.01, and ***p≤0.001. Unless otherwise indicated, all graphical data are reported ±SD.

## Results

### Presentation of matrix proteins following vascular morphogenesis on ECM

**Fig 1:**
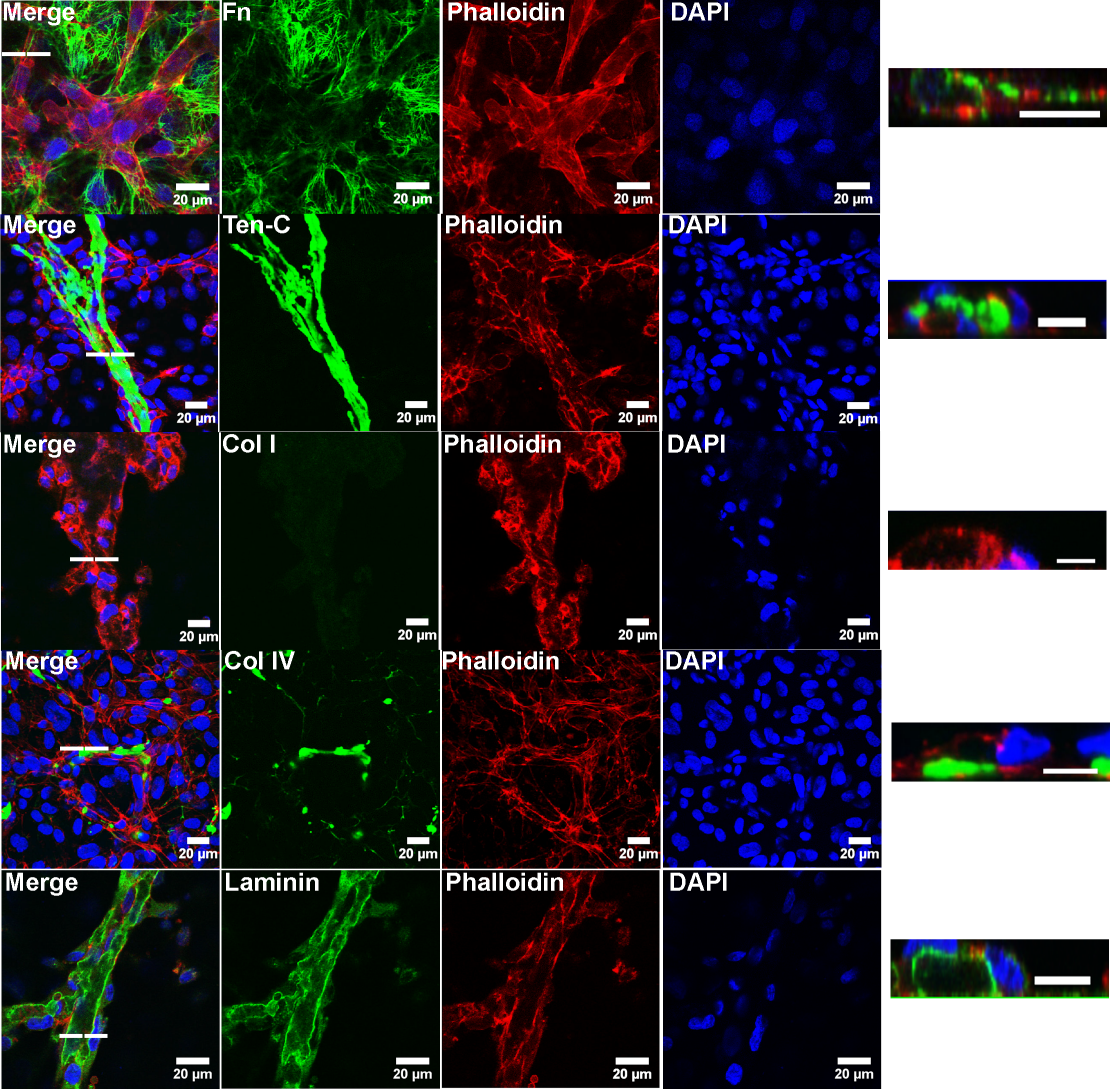
Vascular structures on de-cellularized matrix express high fibronectin. Confocal images of vascular structures illustrate the localization of ECM proteins following vascular morphogenesis of ECs cultured atop de-cellularized co-culture ECM. Images of vascular lumens, indicative of the 3D nature of the structures, were rendered from confocal z stacks. The dashed line indicates the corresponding position of the lumen. Scale bars for cross sectional lumens are 10μM.

ECs were seeded on de-cellularized ECM deposited from co-cultures of dermal fibroblasts (NuFF) with the human breast cancer cell line MDA231 (hereafter co-cultures) and allowed to form vascular structures over a 24 hour period [11, 13, 14]. We elected to employ this co-culture approach as we have previously shown that this culture setup yielded an ECM that was uniformly distributed throughout the culture area, was rich in fibronectin, and supported robust vascular morphogenesis[11]. The average thickness of these de-cellularized matrices was 4.9 ± 0.96μM, a result similar to that reported by Soucy et al [13]. Resulting vascular structures grown on these de-cellularized matrices were fixed and stained for ECM proteins fibronectin, collagens I and IV, tenascin-C and laminin. Out of all ECM proteins evaluated, we found that fibronectin was highly deposited following EC culture atop the de-cellularized matrix, with distinct fibrils located extra-cellularly, bridging the newly formed vascular structures (Fig 1). Deposition of collagen I was negligible, collagen IV was present albeit minimal and laminin was abundant and appeared to be localized to vascular lumens (Fig 1). Tenascin-C was sparsely localized extra-cellularly (Fig 1). Results from these analyses are representative of 3–6 samples for each ECM protein. Low magnification images of vascular structures, illustrating the overall presentation of these ECM proteins following EC culture atop the de-cellularized matrix, are shown in (S1 Fig). These results indicate that patterns of ECM protein localization and deposition, following growth and organization of ECs on the de-cellularized matrices, are unique in comparison to one another.

### MMPs are increased following culture of ECs on de-cellularized ECM

**Fig 2:**
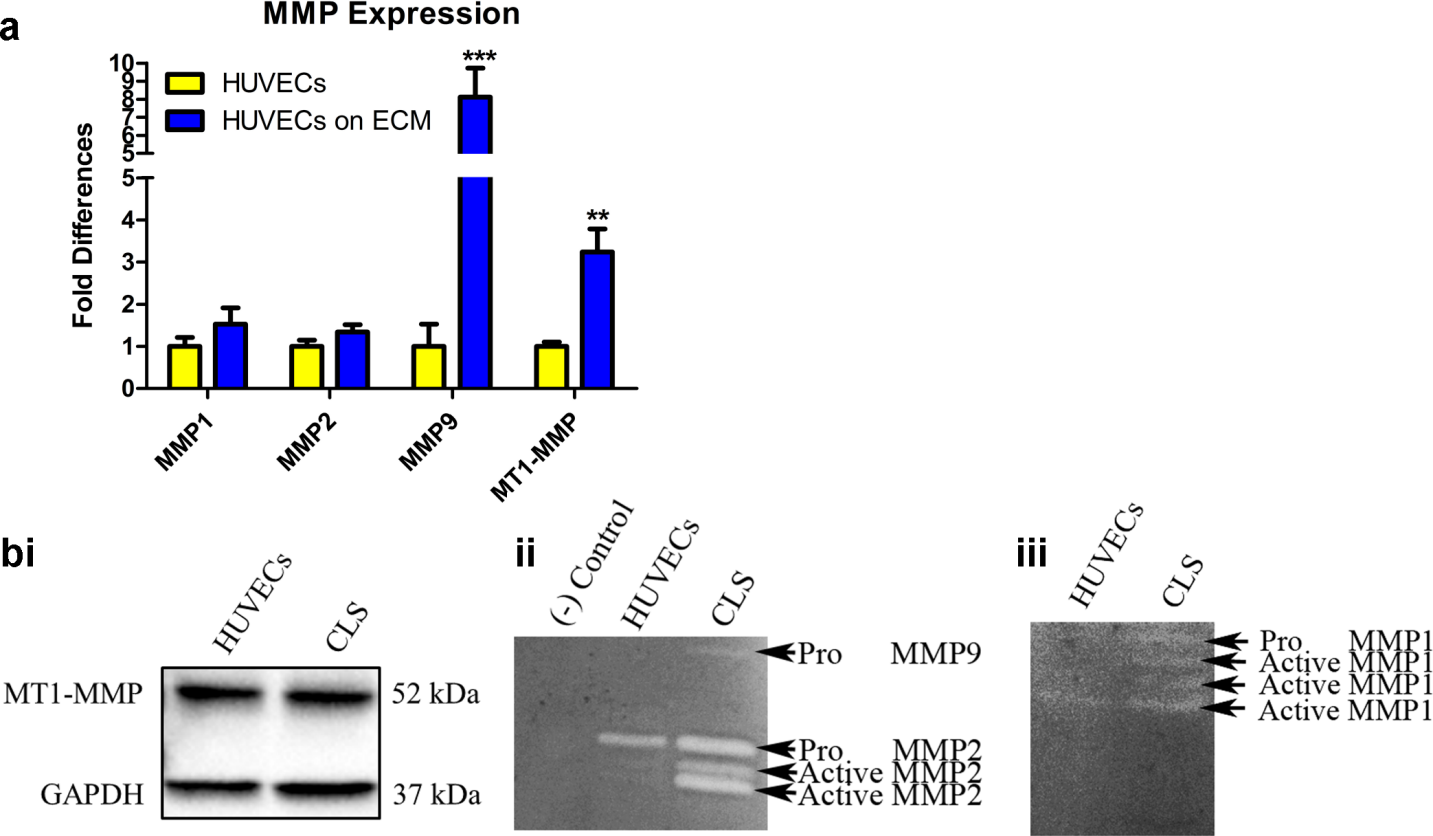
Vascular structures on de-cellularized matrix express active MMPs. **(A)** qRT-PCR illustrated up-regulated expression of MMPs in CLS (ECs on ECM) as compared to ECs cultured on tissue culture plastic. Significance was assessed based on the following p values: *p≤0.05; **p≤0.01; ***p≤0.001. **(Bi)** Western blot did not detect differences in the expression of MT1-MMP between control ECs and CLS. **(Bii)** Enzyme zymography revealed the expression of the active form of MMP2 and the pro-form of MMP9 in CLS. **(Biii)** Active forms of MMP1 were observed in control ECs and CLS although CLS contained additional bands of active forms.

Matrix metalloproteinases (MMPs) play a key role in angiogenesis [19]. Given our observations in which collagens I and IV were negligibly or minimally expressed, respectively, following culture of ECs atop de-cellularized matrices, we elected to evaluate MMPs 1,2, and 9, MMPs which have been shown to degrade collagens 1 and IV [19]. In addition, we also evaluated MT1-MMP, a membrane associated MMP, which activates MMP-2 [20, 21]. We observed up-regulated mRNA expression for all MMPs in ECs cultured for 24 hours on de-cellularized ECM (Fig 2A). While western blot did not reveal differences in MT1-MMP expression between control ECs and vascular structures (Fig 2Bi), zymography demonstrated the presence of MMP-9 and activated forms of MMP-2 in the supernatant collected from vascular structures (Fig 2Bii). Zymography for MMP-1 revealed the presence of several faint, active forms of the enzyme in the supernatant from vascular structures (Fig 2Biii). A faint band representing an active form of MMP-1 was also visible in the supernatant from control ECs cultured on tissue culture plastic dishes (Fig 2Biii). As these analyses were directed at evaluating the presence of the active and inactive forms of the MMPs following culture of ECs on de-cellularized matrices, subtle differences in cell number, a result of differences in cell proliferation, were not anticipated to contribute to any observed differences. Taken together, these results indicate that active MMPs 1,2, and 9, reported by others to degrade collagens I and IV [22–24], are expressed in the supernatant from vascular structures grown atop de-cellularized ECM.

### Fibronectin fibrillogenesis from ECs contributes to vascular morphogenesis

**Fig 3:**
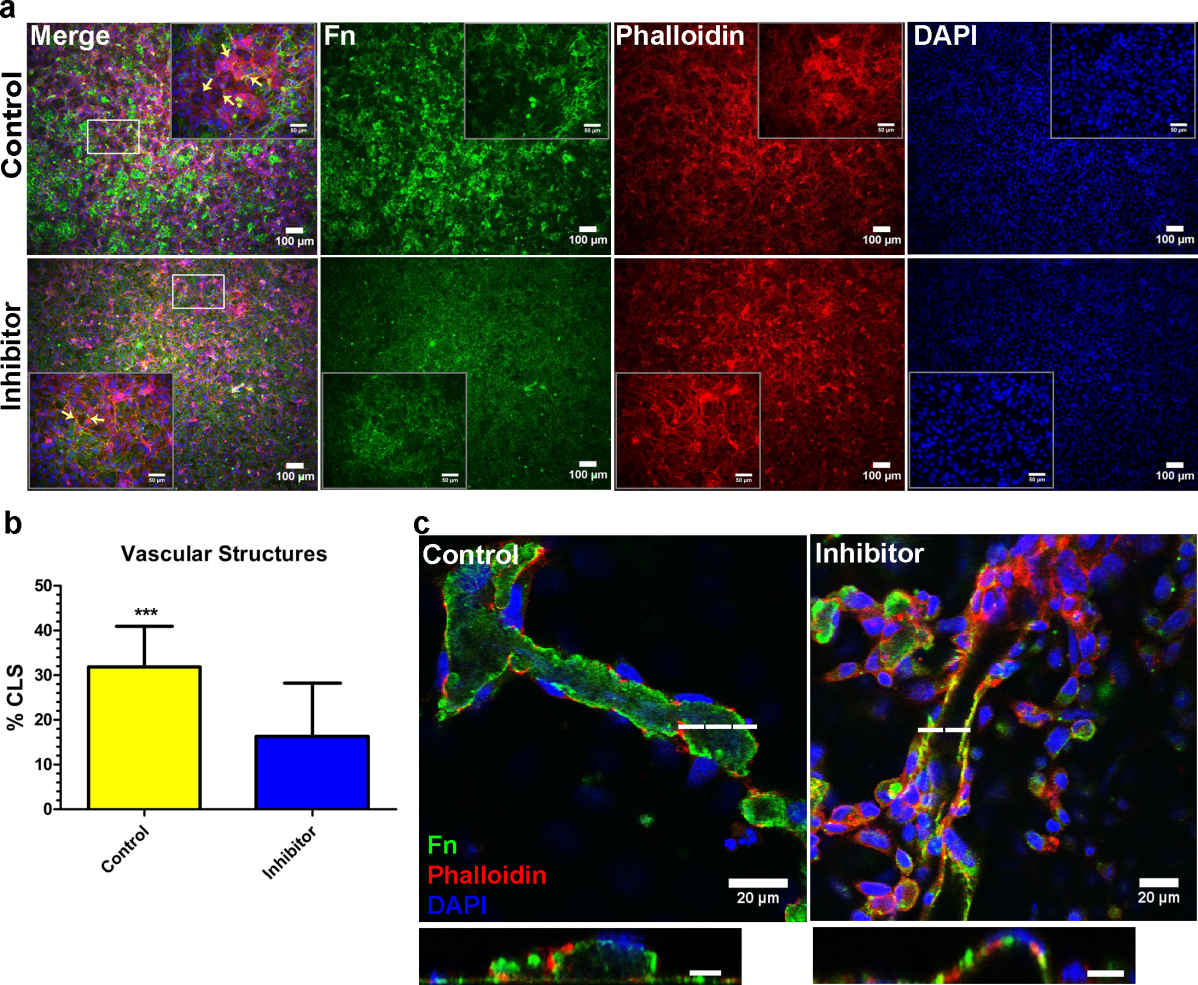
Fibronectin inhibition during vascular morphogenesis reduces CLS formation. **(A)** Representative immunofluorescence images of vascular structures treated with 500μM pUR4B or control, III-11C peptides at the time of EC seeding on ECM. Corresponding high magnification images are shown as insets. Arrows indicate the presence of CLS in high magnification merged inset images. Images for fibronectin were acquired using the same exposure settings. **(B)** Quantification of CLS showed a significantly higher percentage of CLS in structures treated with control III-11C versus pUR4B. **(C)** Confocal images of reconstructed z stacks illustrate the presence of lumens indicative of the 3D nature of the CLS in both control III-11C and pUR4B treated vascular structures. The dashed line indicates the corresponding position of the lumen. Scale bars for cross sectional lumens are 10μM. *p≤0.05; **p≤0.01; ***p≤0.001.

Given the MMP expression analyses, implicating degradation of collagens I and IV in the matrix, and the abundance of fibronectin fibrils from ECs cultured atop de-cellularized matrices, we asked whether EC deposition of fibronectin was responsible for driving patterns of vascular morphogenesis. Prior to these analyses, we investigated the relative abundance of fibronectin in non-cell conditioned media as the serum present in these may be an important source of deposited fibronectin driving vascular morphogenesis. S2A Fig illustrates the abundance of fibronectin in co-culture and EC media. Following these analyses, we sought to inhibit fibronectin fibrillogenesis by ECs during vascular formation. In order to accomplish this, we utilized a small peptide inhibitor of fibronectin polymerization (pUR4B) to inhibit the extracellular assembly of fibronectin from ECs during vascular morphogenesis on de-cellularized co-culture ECM [9, 25]. pUR4B inhibits fibronectin fibrillogenesis by preventing the binding of fibronectin to matrix assembly sites on the surface of cells [25]. As a control, the III-11C peptide, which has no demonstrated activity against fibronectin fibrillogenesis [9, 25], was utilized. We first validated that pUR4B did not decrease fibronectin expression in ECs grown atop de-cellularized co-culture ECM, a result which may otherwise confound our findings. In this manner, ECs were seeded on de-cellularized co-culture ECM and treated with 500nM of pUR4B or control III-11C for 24 hours. After this time, vascular structures were evaluated for changes in fibronectin mRNA and protein expression using qRT-PCR and western blot, respectively. Significant changes in fibronectin mRNA and protein expression were not observed between pUR4B, control III-11C and untreated (e.g. no treatment with pUR4B or III-11C) ECs (S2B and S2C Fig). We then tested whether 250 and 500nM of pUR4B inhibited vascular morphogenesis of ECs on de-cellularized ECM. In this assay, ECs seeded on de-cellularized co-culture ECM were treated with pUR4B or III-11C for 24 hours prior to assessment of luminal capillary like structures (CLS). Immunofluorescence demonstrated reduced CLS following addition of 250nM (data not shown) and 500nM pUR4B to ECs during vascular morphogenesis (Fig 3A). Since luminal CLS were observed to have a higher intensity of pixels than planar sheets of ECs, we were able to quantify the percentage of formed CLS using the threshold function in ImageJ. The average pixel intensity of CLS in III-11C and pUR4B treated ECs was 30.3 ± 5.5 while that of non-CLS was 10.9 ± 2.5. Using this strategy, background fluorescence from sheets of ECs was eliminated, highlighting the CLS which allowed us to quantify percent area occupied by CLS. To confirm that the brighter structures were indeed CLS, we used confocal imaging to generate z stacks. Analyzing z stacks from several confocal images, we documented the presence of lumens in some CLS having higher pixel intensities and the lack of lumens in the dimmer sheets of planar ECs. Quantification of vascular structures revealed a significantly greater percentage of CLS in control, III-11C treated versus pUR4B treated structures (Fig 3B). Representative images from control III-11C and pUR4B treated structures depict the thresh-holding used to quantify the percent CLS (S2D Fig). In an effort to determine whether software equipped to analyze CLS yielded similar findings, we used a previously established method and analyzed CLS formation from III-11C and pUR4B treated ECs grown atop de-cellularized ECM using the angiogenesis function in Metamorph [26–31]. However, results from this analysis yielded inconclusive findings as the software was not sensitive enough to detect the extent of observed vascular formation. This was evidenced by the presence of extensive nodes (green regions) (S3 Fig). Such regions are not classified as vascular structures, but are rather junctions between vascular structures. The presence of vascular structures (white regions) did not appear to correlate with CLS in images thresholded with ImageJ (S3 Fig) that were confirmed to be luminal structures by confocal imaging.

Analysis of extracellular fibronectin revealed a marked decrease in fibronectin expression from pUR4B treated vascular structures as compared to III-11C control (S4 Fig), demonstrating that at the concentration tested, pUR4B reduced fibronectin fibrillogenesis. Despite the reduced number of CLS in pUR4B treated structures, re-constructed z stacks revealed the presence of vascular lumens in pUR4B-treated ECs (Fig 3C), demonstrating that the formed structures were 3D in nature. Taken together, these results suggest that inhibition of fibronectin fibrillogenesis during EC culture atop de-cellularized matrices reduces, but does not eliminate vascular network formation on de-cellularized ECM.

### pUR4B inhibits cellular deposition of a polymerized fibronectin matrix

To address the role of a deposited polymerized fibronectin matrix on vascular morphogenesis, we next asked whether inhibiting fibronectin polymerization in the de-cellularized matrix would alter vascular kinetics and morphogenesis. In order to accomplish this, we utilized pUR4B to inhibit fibronectin polymerization in the de-cellularized co-culture ECM [9, 25]. Co-cultures were treated daily with 250μM of pUR4B or control III-11C over a 9 day period. Representative phase contrast images of pUR4B treated and untreated co-cultures prior to de-cellularization are shown in S5A Fig. The resulting ECM was evaluated for the expression of a polymerized fibronectin matrix. We found that 250μM of pUR4B resulted in complete elimination of a polymerized fibronectin matrix (S5B Fig). In order to exclude the influence of the pUR4B inhibitor on fibronectin expression, we evaluated fibronectin mRNA and protein levels in treated and untreated co-cultures. Results did not reveal a decrease in fibronectin mRNA and protein expression in pUR4B treated co-cultures (S5C and S5D Fig). We further verified that pUR4B did not result in decreased cellular proliferation (S5E Fig). These results illustrate that pUR4B eliminates deposition of a polymerized fibronectin matrix by co-cultures, an observation which is not the result of changes in fibronectin expression or cell proliferation.

### Inhibition of fibronectin fibrillogenesis decreases the deposition of matrix proteins

**Fig 4:**
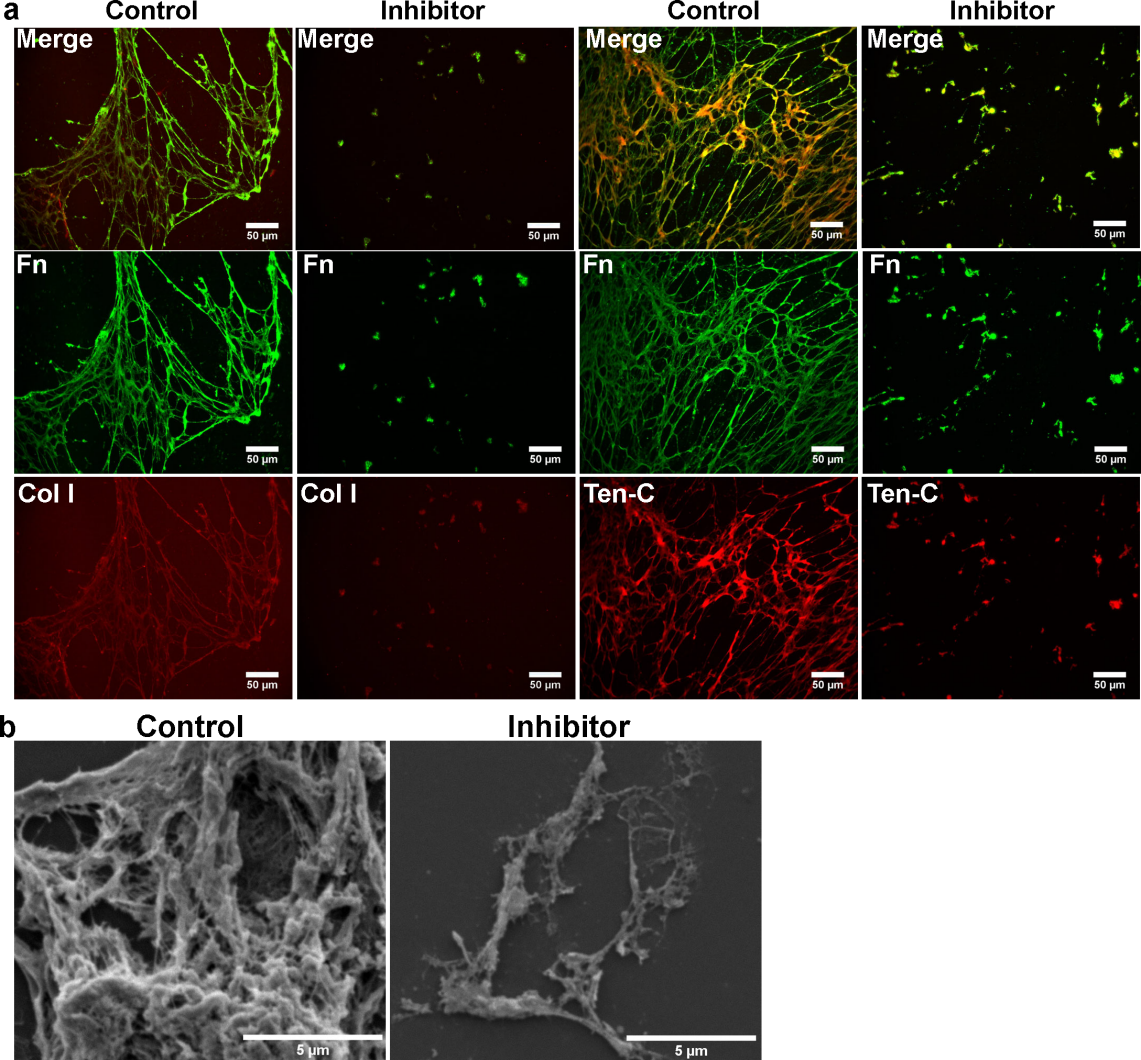
Fibronectin inhibition reduces the deposition of matrix proteins from co-cultures. **(A)** Representative immunofluorescence images of de-cellularized matrix from pUR4B and control III-11C treated co-cultures reveal the absence of collagen I and tenascin-C in co-culture ECM lacking a polymerized fibronectin matrix. **(B)** Ultrastructural high magnification images of ECM deposited by pUR4B and control III-11C treated co-cultures illustrate a marked reduction in the matrix of co-culture ECM lacking a polymerized fibronectin matrix.

We then evaluated whether inhibiting fibronectin polymerization would alter the deposition of other ECM proteins. As described above, co-cultures were treated daily with 250μM of pUR4B or control III-11C over a 9 day period and the resulting ECM evaluated for matrix protein deposition. We observed the absence of ECM proteins collagen I and tenascin-C, matrix proteins known to interact with fibronectin [32], in the de-cellularized matrix from pUR4B treated co-cultures but not from control III-11C treated co-cultures (Fig 4A). These results were not due to alterations in collagen I assembly in the matrix, suggested to be an important factor for fibronectin fibrillogenesis [33], as treatment of NuFF cells with 100nM of halofuginone for 24 hours resulted in the absence of collagen I in regions containing fibronectin fibrils (S6A Fig). Interestingly, we also observed the absence of tenascin-C in control, untreated NuFF cultures despite the presence of extracellular fibronectin fibrils (S6B Fig), suggesting that tenascin-C matrix assembly does not precede fibronectin fibrillogenesis in the matrix. We further evaluated the de-cellularized matrix from pUR4B treated co-cultures using scanning electron microscopy, a measure allowing us to observe all deposited matrix proteins. Results revealed little ECM deposited from pUR4B treated co-cultures in comparison to control co-cultures (Fig 4B), confirming that the absence of a polymerized fibronectin matrix markedly reduces the assembly of other matrix proteins. These results suggest that a polymerized fibronectin matrix serves as a substrate from which other ECM proteins bind.

### Matrix proteins are highly co-localized with the fibronectin matrix

**Fig 5:**
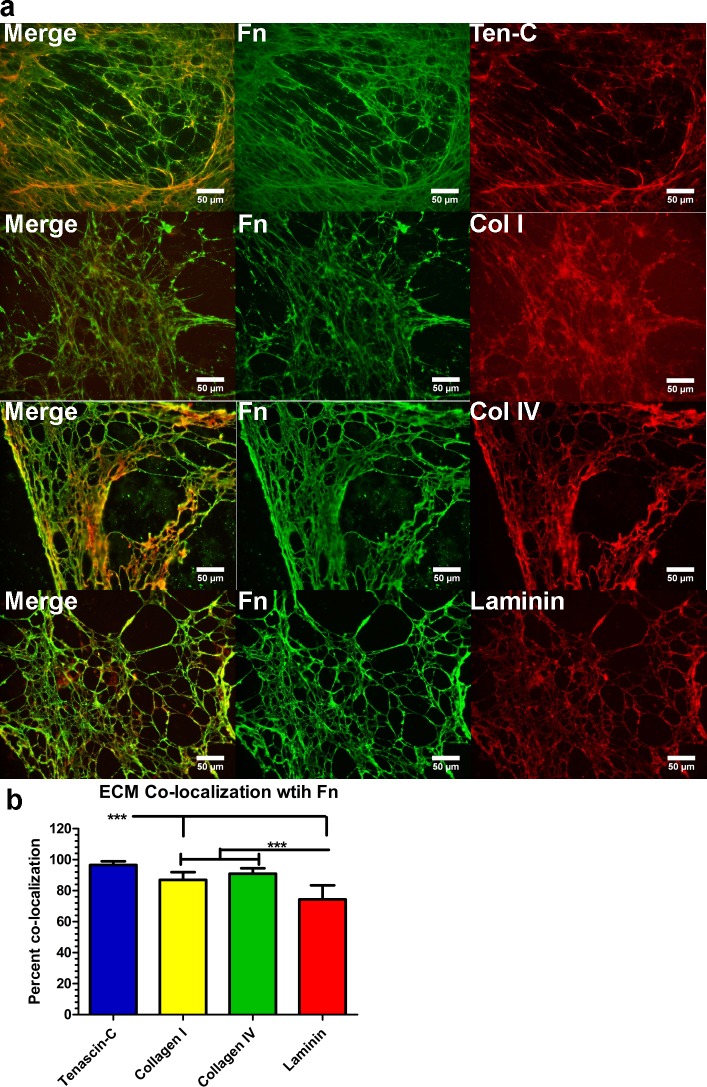
Matrix proteins are highly co-localized with a polymerized fibronectin matrix. **(A)** Representative immunofluorescence images of matrix proteins co-localized with the polymerized fibronectin matrix show co-localization of collagens I and IV, laminin and tenascin-C with fibronectin. **(B)** Quantification of matrix proteins co-localized with the polymerized fibronectin matrix. Fn: fibronectin; Ten-C: tenascin-C; Col I, IV; collagens I or IV. *p≤0.05; **p≤0.01; ***p≤0.001.

Intrigued by the results wherein the loss of a polymerized fibronectin matrix led to a concomitant loss in collagen I and tenascin-C and marked reduction in general matrix expression, we further investigated the degree to which several matrix proteins are co-expressed with fibronectin in the matrix. We noticed that collagens I and IV, tenascin-C and laminin were highly co-localized to the fibronectin matrix deposited by untreated co-cultures (Fig 5A). Similar to observations from pUR4B treated co-cultures, we observed that in regions lacking a polymerized fibronectin matrix, there was a concomitant absence of additional matrix protein expression (data not shown). While each of the ECM proteins tested were highly co-localized with fibronectin, the greatest degree of co-localization was observed for tenascin-C (Fig 5B). In order to determine whether these results were not due to affects from culture conditions (e.g. co-culturing), we evaluated co-localization of ECM proteins with fibronectin deposited by NuFF cells. Similarly, we observed a high degree of ECM protein co-localization with the polymerized fibronectin matrix deposited by NuFF cells (data not shown). These data not only confirm prior reports of collagen I and tenascin-C interaction with a fibronectin matrix [32], but additionally suggest that fibronectin may serve as a scaffold directing the assembly of several matrix proteins.

### ECs exhibit altered migration in the absence of a polymerized fibronectin matrix

**Fig 6:**
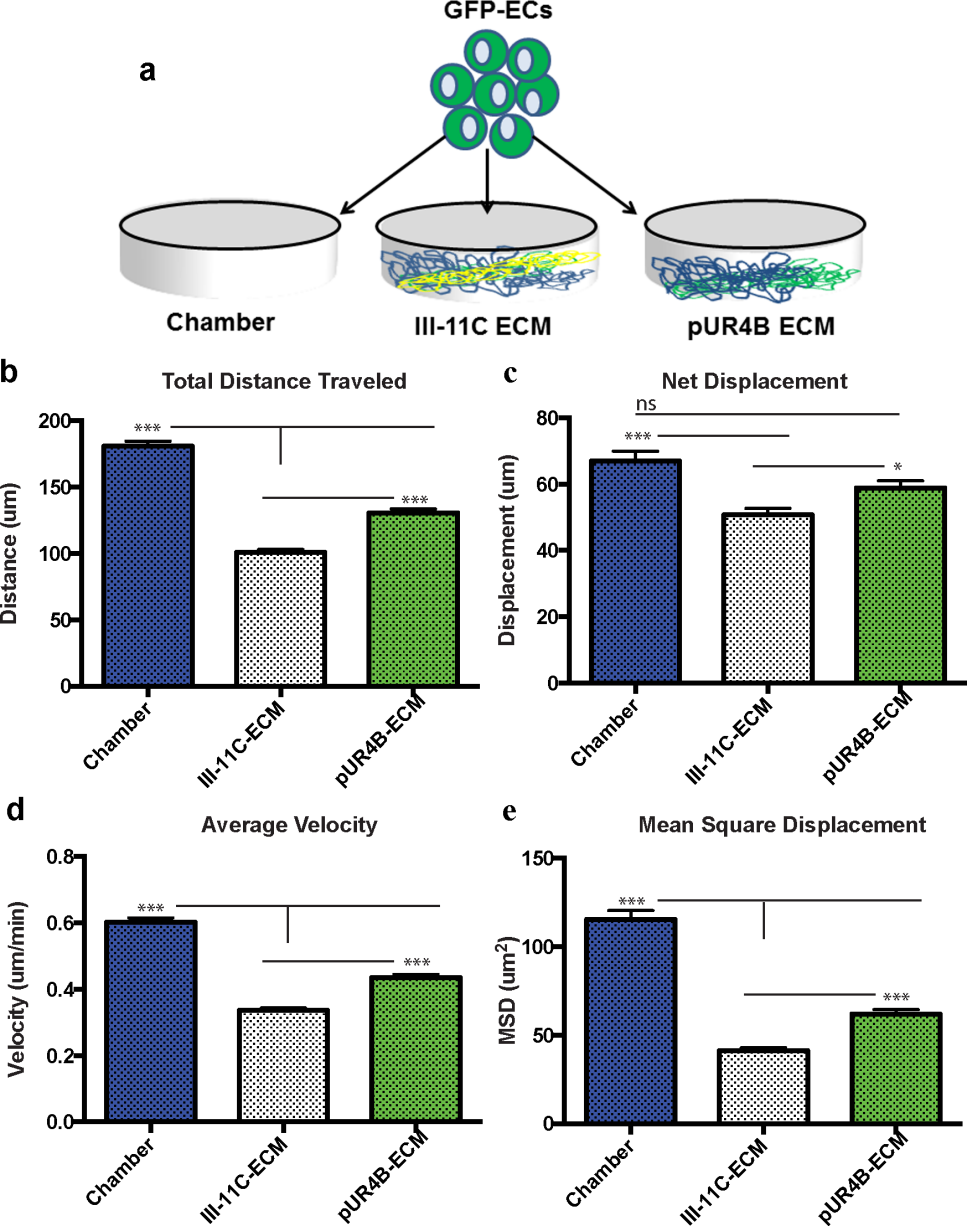
Vascular kinetics is altered following culture of GFP+ ECs atop pUR4B or III-11C ECM. **(A)** GFP+ ECs were seeded on Chambers, III-11C-ECM and pUR4B-ECM and were assessed for differences in vascular kinetics between hours 7 and 12 post-seeding. Graphs depict **(B)** total distance traveled, **(C)** net displacement, **(D)** average velocity, and **(E)** mean square displacement of the GFP+ ECs. *p≤0.05; **p≤0.01; ***p≤0.001. The error bars represent the SEM.

To assess how the polymerized fibronectin matrix affects vascular kinetics, we cultured GFP transfected ECs (S7A Fig) atop ECM from pUR4B treated (pUR4B-ECM), control III-11C treated (III-11C-ECM) and un-coated chamberslides (Chambers) at hour 0. A schematic of this set up is shown in Fig 6A. All analyses were performed on ECs cultured in media containing 2% serum. Live cell time-lapse images were used to track EC migration and were taken at 15-minute intervals following their attachment. We obtained the coordinates of the positions of the cells at each time point where the center of the tracked cells was used as a reference point for each frame. Cells that entered or exited the field of view during the 5-hour tracking time interval were excluded and cells that underwent apoptosis or proliferation were also disregarded during the tracking process. Between 60 and 100 cells were tracked per condition per experiment. Each experiment was repeated three times and each condition was performed in duplicate. S7B Fig shows the trajectories of ECs in each of the tested conditions after 5 hours of imaging. Of all tested conditions, ECs seeded on the Chambers migrated the greatest distance (180.9μm) while the ECs on III-11C-ECM traveled the least distance (100.9 μm) (Fig 6B). Interestingly, ECs on pUR4B-ECM migrated a mean distance of 130.8 μm, a value which was significantly less than ECs on Chambers, but significantly greater than ECs on III-11C-ECM (Fig 6B).

In addition to the total distance migrated, we also calculated the net displacement of ECs during the 5 hour imaging period. Net displacement is the measurement of the distance from a cell’s original position at t = 0 to its final position at t = 300 minutes. This parameter only takes into consideration the first and last positions, whereas the total distance traveled takes into consideration all distances traveled by a cell between t = 0 to t = 300 minutes. Similar to the previous results, EC net displacement was greatest on the Chambers with a mean value of 67.00 μm (Fig 6C). Similarly, ECs on pUR4B-ECM had a net displacement of 58.93μm (Fig 6C). These values are significantly greater than the net displacement of ECs on III-11C-ECM (50.79μm) (Fig 6C).

Another kinetic parameter we quantified was the average velocity of ECs on each of the tested conditions. Fig 6D shows the average velocity of the ECs on each of the tested conditions for the 5 hour imaging period. ECs migrated at the highest rate on the Chambers with an average velocity of 0.603μm/min (Fig 6D). The second highest velocity was observed from ECs on pUR4B-ECM, which migrated at a rate of 0.436μm/min (Fig 6D**)**. These values were significantly higher than ECs on III-11C-ECM, which had an average velocity of 0.336 μm/min (Fig 6D).

Finally, we measured the mean square displacement (MSD) of ECs on each of the tested conditions. This parameter permits a better understanding of how cell migration dynamics relate to the mechanical properties of the substrate[34]. Fig 6E depicts the MSD for each tested condition and represents data collected for the 5 hour imaging period. ECs on the Chambers exhibited the highest MSD with a value of 115.5 μm^2^ (Fig 6E). This was followed by ECs on pUR4B-ECM, which had a MSD of 61.91 μm^2^ (Fig 6E). MSDs for the Chamber and pUR4B-ECM were significantly higher in comparison to III-11C-ECM, which exhibited a MSD of 41.34 μm^2^ (Fig 6E). Our data suggest that a polymerized fibronectin matrix modulates vascular kinetics.

### Vascular organization is absent on ECM without a polymerized fibronectin matrix

To address how polymerized fibronectin contributes to vascular morphogenesis, we cultured ECs on de-cellularized co-culture ECM with or without a polymerized fibronectin matrix. In this analysis, GFP-ECs were seeded on pUR4B-ECM, III-11C-ECM or Chambers as detailed above. After 12 hours, ECs seeded on the Chambers exhibited no evidence of vascular organization, apparent as the cells have a sheet-like morphology with absence of any nuclear alignment into branched configurations (S8 Fig). A similar morphology was observed for ECs on pUR4B-ECM, indicative of the absence of vascular organization (S8 Fig). In contrast, there was some vascular organization observed for the ECs seeded on III-11C-ECM, evident from the alignment of nuclei into branched configurations (S8 Fig). These data suggest that vascular organization takes place on de-cellularized ECM in which a polymerized fibronectin matrix is present.

### Integrins α_5_β_1_ and α_v_β_3_ are indispensable for vascular morphogenesis of ECs on de-cellularized ECM

**Fig 7:**
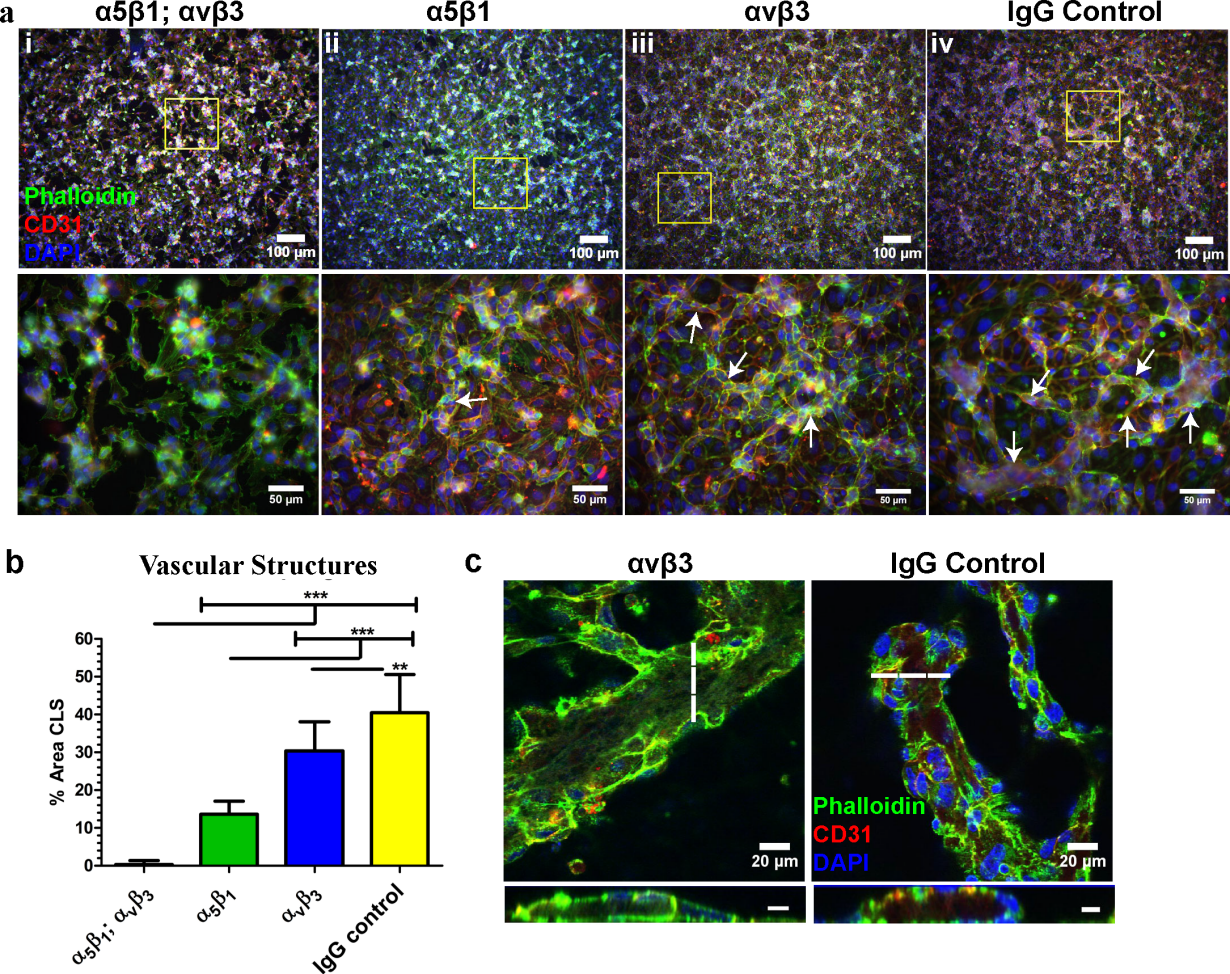
Inhibition of vascular integrin adhesion to a polymerized fibronectin matrix disrupts CLS formation. **(A)** Representative immunofluorescence images of vascular structures treated with blocking antibodies to the fibronectin integrins α_5_β_1;_α_v_β_3,_ α_5_β_1_, and α_v_β_3_ and control IgG. *Top panel*; low magnification. *Bottom panel*; high magnification. Vascular structures are indicated with white arrows. **(B)** Quantification of CLS revealed near absent vascular morphogenesis in ECs treated with anti-α_5_β_1;_α_v_β_3_ antibodies while ECs treated with control IgG antibody possessed the greatest percentage of CLS. **(C)** Vascular lumens were present in structures treated with anti-α_v_β_3_ and IgG control antibody only. The dashed line indicates the corresponding position of the lumen. Scale bars for cross sectional lumens are 10μM. *p≤0.05; **p≤0.01; ***p≤0.001.

Since patterns of vascular kinetics and organization were altered on ECM lacking polymerized fibronectin, we asked whether vascular morphogenesis would be disrupted in ECs, which could not adhere to the fibronectin matrix. To address this question, we treated ECs with blocking antibodies against the fibronectin integrins α_5_β_1_ and α_v_β_3_ prior to seeding on de-cellularized co-culture ECM. The combination of α_5_β_1_ and α_v_β_3_ resulted in almost complete inhibition of vascular morphogenesis (Fig 7Ai and 7B) while α_5_β_1_ block alone significantly reduced vascular morphogenesis (Fig 7Aii and 7B) and α_v_β_3_ block slightly retarded vascular morphogenesis (Fig 7Aiii and 7B) in comparison to IgG control (Fig 7Aiv and 7B). Evidence of vascular lumens were seen for α_v_β_3_ and control IgG treated ECs (Fig 7C), indicating that the structures assembled into a 3D vascular network. Vascular lumens could not be identified for α_5_β_1_/α_v_β_3_ and α_5_β_1_ treated ECs on de-cellularized ECM (data not shown). These results indicate a role for a polymerized fibronectin matrix in the directed assembly of vascular structures via integrins α_5_β_1_ and α_v_β_3_.

## Discussion

A key aspect underlying both regenerative medicine and the field of cancer research is a more complete understanding of how the cell’s microenvironment regulates angiogenesis. In this manner, numerous groups have utilized 2D ECM coated surfaces or 3D matrices of natural (e.g. fibrin or collagen) or synthetic sources of ECM mimetic materials to achieve these goals. Undoubtedly, these studies have provided great depth of insight into how the composition of the ECM in addition to its physical and mechanical attributes regulate cell behaviors. In this study, we have attempted to gain a better understanding of how a purely biological, fibronectin-rich matrix deposited from cells directs vascular morphogenesis. Overall, we found that fibronectin is critical for vascular morphogenesis and matrix assembly *in-vitro*.

In an attempt to characterize fibronectin presentation in vascular structures formed atop de-cellularized ECM, we utilized immunofluorescence and confocal imaging to detect the overall presentation of fibronectin in addition to other ECM proteins having known roles in angiogenesis [35]. We observed negligible to minimal expression of collagens I and IV, respectively, discovering increased expression of the collagenase MMP1 and the gelatinases MMPs 2 and 9 in ECs cultured atop de-cellularized matrices. Tenascin-C was primarily localized to the extra-luminal surface of formed vascular structures, confirming a report by Berndt et al [36] who documented extra-luminal expression of tenascin-C in the vessels of tumors. While the functional significance of this localization pattern is unknown, it is interesting to speculate that tenascin-C may act to stabilize newly formed vessels. The ECM proteins most abundant following culture of ECs atop de-cellularized matrices were fibronectin and laminin. Laminin was primarily localized to the luminal surface of vascular structures, a puzzling observation as it is predominantly localized in the basement membrane. Although unexpected, others have also reported an apical expression of laminin. Wautier et al [37] reported localization of laminin α5 to the apical surface of HUVECs while Hillario et al [38] similarly observed localization of laminin and the 67 kDa laminin receptor to the luminal surface of lung capillaries. It’s possible that the luminal localization of laminin may help support vascular maturation, a function of laminin reported by Thyboll et al [39]. Fibronectin, on the other hand, extensively enveloped the newly formed vascular structures, implicating a potentially crucial role for this matrix protein in directing vascular network assembly. Unfortunately, we could not differentiate EC-produced fibronectin versus fibronectin pre-deposited in the de-cellularized matrix prior to vascular morphogenesis. In future studies, it will be important to address the contributions from each of these cell populations.

Vascular morphogenesis has previously been shown to be directed by fibronectin fibrillogenesis of ECs grown in 3D fibrin gels [8]. Given these results in addition to our observations, we sought to determine whether fibronectin deposition by ECs cultured on ECM was similarly responsible for directing vascular morphogenesis. To test this, we abolished fibronectin fibrillogenesis through use of a small peptide, pUR4B, which interferes with fibronectin polymerization in the matrix [8, 9, 25]. We demonstrated that addition of pUR4B to ECs at the time of seeding on de-cellularized ECM significantly decreased vascular morphogenesis in comparison to control III-11C treated ECs. These results are in line with prior reports which indicate that fibronectin fibrillogenesis is indispensable for neovessel formation in 3D fibrin gels [8]. While pUR4B significantly disrupted vascular morphogenesis, it did not completely inhibit vascular morphogenesis of ECs on ECM. It is unlikely that a higher concentration of pUR4B may have achieved a greater effect on vascular morphogenesis as concentrations of 250 and 500nM gave similar results. As a result, it’s reasonable to conclude that while pUR4B significantly disrupts fibronectin fibrillogenesis resulting in reduced vascular morphogenesis of ECs cultured atop de-cellularized ECM, the peptide may not completely block all fibronectin assembly. To determine whether our results were in line with data generated using commercially available software, we utilized the angiogenesis function in Metamorph as previously reported [26–31]. Upon evaluating all images from pUR4B and III-11C treated ECs cultured atop de-cellularized ECM, we observed that the sensitivity of this program to detect CLS in our culture setup was low. This was apparent given the extensive presence of nodes or vascular junctions and regions classified as vascular structures which did not correlate with the observed patterns of CLS organization in regions with higher pixel intensity. Given that the algorithm is best suited for analyzing CLS from ECs cultured on Matrigel or within a hydrogel matrix, a platform which yields robust CLS, it’s likely that the software is not suitable for identifying CLS amongst other non-CLS. In this manner, the complexity of CLS formed in our culture setup is not suitable for analysis using the angiogenesis function in Metamorph. While we believe that we can reliably characterize CLS based on pixel intensity differences, we recognize that better imaging strategies are necessary to identify and quantify CLS formed atop de-cellularized ECM. Together, these results confirm an indispensable role for EC-produced fibronectin in vascular morphogenesis of ECs cultured atop de-cellularized ECM. In future studies, it will be important to address differences in vascular morphology, such as tube length and branching patterns, in structures formed atop de-cellularized ECM in the presence and absence of pUR4B.

Given these findings, our next step was to address the role of the polymerized fibronectin matrix on vascular morphogenesis. Previous work in our lab has demonstrated that co-cultures of NuFF with MDA cells elaborated a rich fibronectin matrix [11]. With these former results in mind, we treated co-cultures of NuFF and MDA cells with pUR4B and characterized the deposited de-cellularized ECM for evidence of fibronectin polymerization. We observed absent fibronectin polymerization in the matrix of co-cultures treated with pUR4B, but not in cultures treated with control III-11C. The absence of fibronectin resulted in loss of tenascin-C and collagen I fibers, two proteins known to interact with fibronectin [32]. Upon further examination using ultrastructural analyses, we observed a marked decrease in matrix proteins in the de-cellularized ECM from pUR4B treated co-cultures. Although the identities of these proteins are unknown, they are most likely residual fibrillar proteins given their morphology. Importantly, we demonstrate that these results were not due to collagen I, reported to be a prerequisite for fibronectin binding in the matrix [33]. Considering these observations, we evaluated the extent to which other ECM proteins are co-deposited with a polymerized fibronectin matrix in the de-cellularized ECM. Previously, it has been reported that fibronectin co-localizes with collagen I, tenascin-C, fibrin and thrombospondin [12, 32]. Previous recent findings have reported that fibronectin polymerization is necessary for assembly of collagen I [12, 40–42] and thromospondin [12], highlighting the important role of fibronectin in matrix assembly. As expected, we observed a high degree of co-localization of collagen I and tenascin-C with fibronectin, collagen IV and laminin with fibronectin in co-culture ECM. Since binding sites for collagen IV and laminin on fibronectin have not been reported, it’s possible that these proteins may indirectly associate with fibronectin through other matrix proteins. Combined, these results not only indicated that pUR4B inhibited fibronectin polymerization in the de-cellularized matrix, but additionally pointed to a role for polymerized fibronectin in the directed assembly of other matrix proteins.

Fibronectin has previously been shown to promote EC migration [5, 6, 43]^,^[44, 45]. To address the role of a biological, 3D polymerized fibronectin matrix on vascular kinetics, we seeded ECs atop co-culture ECM lacking a polymerized fibronectin matrix. We discovered that migration and velocity of migration were highest for ECs cultured in the absence of both an intact ECM (e.g. Chambers) and a polymerized fibronectin matrix (e.g. pUR4B-ECM). EC migration and velocity of migration were lowest on matrices containing a polymerized fibronectin matrix (e.g. III-11C-ECM). Since our studies were performed using a 3D matrix as opposed to a 2D matrix, we speculate that the presence and organization of the fibronectin fibrils in the matrix may have played a role in these observations. However, given the marked reduction of ECM from pUR4B treated co-cultures, we cannot rule out the possibility that some of the observed effects of pUR4B-ECM on vascular behavior were a result of regions lacking a polymerized matrix. In addition, given our observations where EC migration was greatest in the absence of an intact ECM and on pUR4B-ECM and lowest on matrices containing polymerized fibronectin, it is likely that EC migration may be optimal at intermediate matrix concentrations and/or compositions. For example, Zaman et al [46] used a computational approach to illustrate that cell migration is maximized in 3D matrices with intermediate stiffness and ligand density. With regard to ECs, it was shown that EC attachment, spreading and vascular network formation [47] and EC migration [48] is optimal on ECM matrices of intermediate density, observations which were subsequently validated computationally by Bauer et al [49]. It is apparent that matrix density is an important feature regulating cell migration and in future studies, it will be necessary to investigate whether the topographical and viscoelasticity features of fibronectin relate to cell migratory behavior.

Previous work in our lab has shown that fibronectin patterning of 2D surfaces guided the attachment and elongation of endothelial progenitor cells [7] while fibronectin patterning of 3D micropillars similarly promoted EC attachment and alignment [50]. To address the role of a polymerized fibronectin matrix on patterns of vascular organization, we seeded ECs atop co-culture ECM lacking a polymerized fibronectin matrix. We found that vascular organization, evidenced as the presence of nuclear alignment and cellular organization, was absent for ECs cultured on ECM lacking a polymerized fibronectin matrix. Vascular organization was, however, present on ECM containing a polymerized fibronectin matrix and were evident as early as 12 hours post-seeding. In all, these results support a role for polymerized fibronectin in the directed assembly of vascular-like structures.

Integrins are transmembrane receptors which facilitate cellular interactions with the environment. In particular, α_5_β_1_ and α_v_β_3_ have not only been shown to bind to the fibronectin matrix [4, 51–53], but have also been reported to facilitate EC activation and proliferation through interactions with fibronectin [4]. As such, we evaluated whether vascular morphogenesis could be disrupted in ECs which could not bind to the fibronectin matrix. Using blocking antibodies against α_5_β_1_ and α_v_β_3_ integrins, we found that CLS from ECs cultured on de-cellularized ECM was almost completely abolished. Blocking α_5_β_1_ and α_v_β_3_ separately also significantly reduced CLS, but not to the same extent as that observed for both α_5_β_1_ and α_v_β_3._ These results suggest that the combination of α_5_β_1_ and α_v_β_3_ is crucial for vascular morphogenesis of ECs on de-cellularized ECM and complement work by others who have reported that α_5_β_1_ and α_v_β_3_ were necessary for vascular morphogenesis of ECs suspended in a 3D fibrin gel [18]. We do not believe our results stem from anti-integrin mediated effects on the interaction of ECs with the de-cellularized matrix as no detectable differences in the presence of non-adhered cells (e.g. cells floating in the media) were observed in anti-integrin treated ECs in comparison to non-treated ECs. We recognize that α_v_β_3_ also binds to vitronectin [54]. Although it will be important to delineate a potential contribution for vitronectin in vascular morphogenesis of ECs on de-cellularized ECM, we believe that fibronectin is the major player. Given our above findings we speculate that both α_5_β_1_ and α_v_β_3_ integrins facilitate vascular morphogenesis through interactions with polymerized fibronectin in the de-cellularized ECM. Moreover, we believe that α_v_β_3_ may play a lesser role in vascular morphogenesis overall as blocking with α_5_β_1_ markedly reduced vascular morphogenesis. Considering our initial results, we propose that ECs must first attach to a polymerized fibronectin matrix prior to vascular network assembly, after which time EC-derived fibronectin fibrillogenesis helps drive vascular morphogenesis.

## Conclusions

Overall, we report for the first time that a completely biological, cell derived polymerized fibronectin matrix is indispensable for vascular morphogenesis and stromal matrix assembly. The novelty of these studies lies in the use of a purely biological 3D cell-derived ECM, as opposed to 2D cultures or 3D hydrogels of one protein (e.g. fibrin, collagen), from which to investigate vascular morphogenesis and matrix assembly dynamics. As such, we have shown, using a small peptide inhibitor of fibronectin fibrillogenesis, that we can de-couple the contribution of a polymerized fibronectin matrix in vascular morphogenesis and matrix assembly from a myriad of additional matrix proteins present in the de-cellularized 3D ECM. In this manner, we believe these studies provide a more complete understanding of the micro-environmental factors regulating vascular morphogenesis, knowledge that may be utilized for the construction of sophisticated scaffolds designed to promote angiogenesis during tissue healing and regeneration. In addition, these results may also translate to the field of cancer research. Since fibronectin is a feature of solid tumors and their vessels [55–57], this research not only opens up opportunities for investigation of a potential role for fibronectin in tumor initiation and angiogenesis, but may additionally lead to the future development of agents which disrupt cancer and vascular cell interactions with a fibronectin matrix during tumorigenesis. Finally, these studies promote opportunities for better replicating the 3D in-vivo environment. For instance, future studies could investigate how ECs, sandwiched between layers of ECM, undergo vascular morphogenesis in response to matrix cues presented to both the basal and apical surfaces of the ECs.

## Supporting Information

S1 Fig Vascular structures grown atop de-cellularized matrix express several ECM proteins. Low magnification immunofluorescence images of vascular structures depict the overall presentation of ECM proteins collagens I and IV, tenascin-C and fibronectin following vascular morphogenesis of ECs on de-cellularized co-culture ECM.
(TIF)

S2 Fig Fibronectin inhibition does not significantly affect fibronectin gene and protein expression in ECs following vascular morphogenesis. **(A)** Baseline fibronectin expression in co-culture and EC media. Results show fibronectin expression in the absence of cell produced fibronectin. **(B)** qRT-PCR and **(C)** western blot of fibronectin expression in ECs treated with pUR4B and control III-11C peptides at the time of seeding on de-cellularized co-culture ECM. *p≤0.05; **p≤0.01; ***p≤0.001. **(D)** Images of phalloidin-stained vascular structures before and after threshold using ImageJ. Thresholded images were used for analysis of the percent area occupied by CLS. Arrows indicate the presence of CLS in non-thresholded images.
(TIF)

S3 Fig CLS analyses using the angiogenesis tool in Metamorph. Images of CLS were generated using the angiogenesis tool in Metamorph. Images are from pUR4B (inhibitor) and control III-11C treated ECs cultured for 24 hours atop de-cellularized ECM matrices. The white regions are indicative of vascular structures while the green regions are indicative of nodes or vascular junctions. These nodes occupy a majority of the highlighted regions in both images. For comparison, the same images are shown before (*top panel*) and after thresholding (*bottom panel*), which was used to identify structures based on differences in pixel intensity.
(TIF)

S4 Fig Fibronectin inhibition reduces the intensity of fibronectin expression in vascular structures. Confocal images of vascular structures treated with pUR4B and control III-11C peptides at the time of seeding on de-cellularized ECM. Images for pUR4B and control III-11C illustrate fibronectin expression in the first and last 3 sets of z stacks for vascular structures. Image 1 corresponds to the top of the well while image 6 corresponds to the bottom of the well. The intensity of fibronectin expression was greatest for vascular structures treated with control III-11C and lowest for vascular structures treated with the pUR4B inhibitor. Images were obtained using the same microscope settings.
(TIF)

S5 Fig Fibronectin inhibition eliminates fibronectin fibrillogenesis in the co-culture matrix but does not alter fibronectin gene or protein expression. **(A)** Representative phase contrast images of pUR4B treated and untreated NuFF/MDA231 co-cultures prior to de-cellularization. **(B)** Representative immunofluorescence images of fibronectin in de-cellularized ECM from untreated, control III-11C treated and pUR4B treated co-cultures. **(C)** qRT-PCR and **(D)** western blot of fibronectin expression in control III-11C treated and pUR4B treated co-cultures. **(E)** Analysis of cell proliferation in untreated, control III-11C treated and pUR4B treated co-cultures. *p≤0.05; **p≤0.01; ***p≤0.001.
(TIF)

S6 Fig Inhibition of collagen I deposition does not alter fibronectin fibril formation in co-cultures. **(A)** Representative immunofluorescence images of NuFF cells treated with 100nM of halofuginone for 24 hours. Collagen I fibrils are observed with fibronectin fibrils in control NuFF cells, but are absent in halofuginone treated NuFF despite the presence of extracellular fibronectin fibrils. **(B)** Immunofluorescence images of control NuFF cells illustrate the absence of tenascin-C where fibronectin fibrils are observed. HF: Halofuginone.
(TIF)

S7 Fig Final trajectories of GFP+ ECs seeded on the various treatment conditions. **(A)** ECs were transduced with lentiviral GFP and were sorted into GFP+ and GFP- subpopulations. Corresponding phase contract and fluorescence images were taken of both populations. These images depict the high fluorescence observed for the GFP+ subpopulation and the lack of fluorescence observed for the GFP+ subpopulation. Scale bars = 100μM. **(B)** The migration of the GFP+ ECs was monitored by tracking their positions every 15 minutes for a total of 5 hours. These images show the cells’ trajectories after the 5-hour time period. The *top panel* depicts trajectories with cells and the *bottom panel* depicts trajectories without the cells. The ECs are in grey scale while different colored lines represent the trajectories of random selected cells. The 5-hour trajectories were used to determine the total distance traveled by the ECs on each of the scaffolds.
(TIF)

S8 Fig Inhibition of fibronectin fibrillogenesis in the de-cellularized matrix prevents vascular organization. Confocal images were taken of GFP+ EC organization following growth on Chambers, III-11C-ECM and pUR4B-ECM. All images were acquired 12 hours post-seeding. Differences in vascular organization were evident from each of the tested conditions. ECs on Chambers and pUR4B-ECM have a sheet-like morphology with no evidence of nuclear alignment. ECs on III-11C-ECM exhibit vascular organization, evident by the presence of nuclear alignment into branched-like structures. These organized structures are indicated with white arrows. Scale bars = 100μM.
(TIF)

S1 Table List of antibodies and dilutions used in study. (DOCX)
