# Supplementary figures and images for: Fibronectin Deposition Participates in Extracellular Matrix Assembly and Vascular Morphogenesis
Source: PLoS One. 2016 Jan 26;11(1):e0147600. doi: 10.1371/journal.pone.0147600 (PMC4728102; doi:10.1371/journal.pone.0147600)

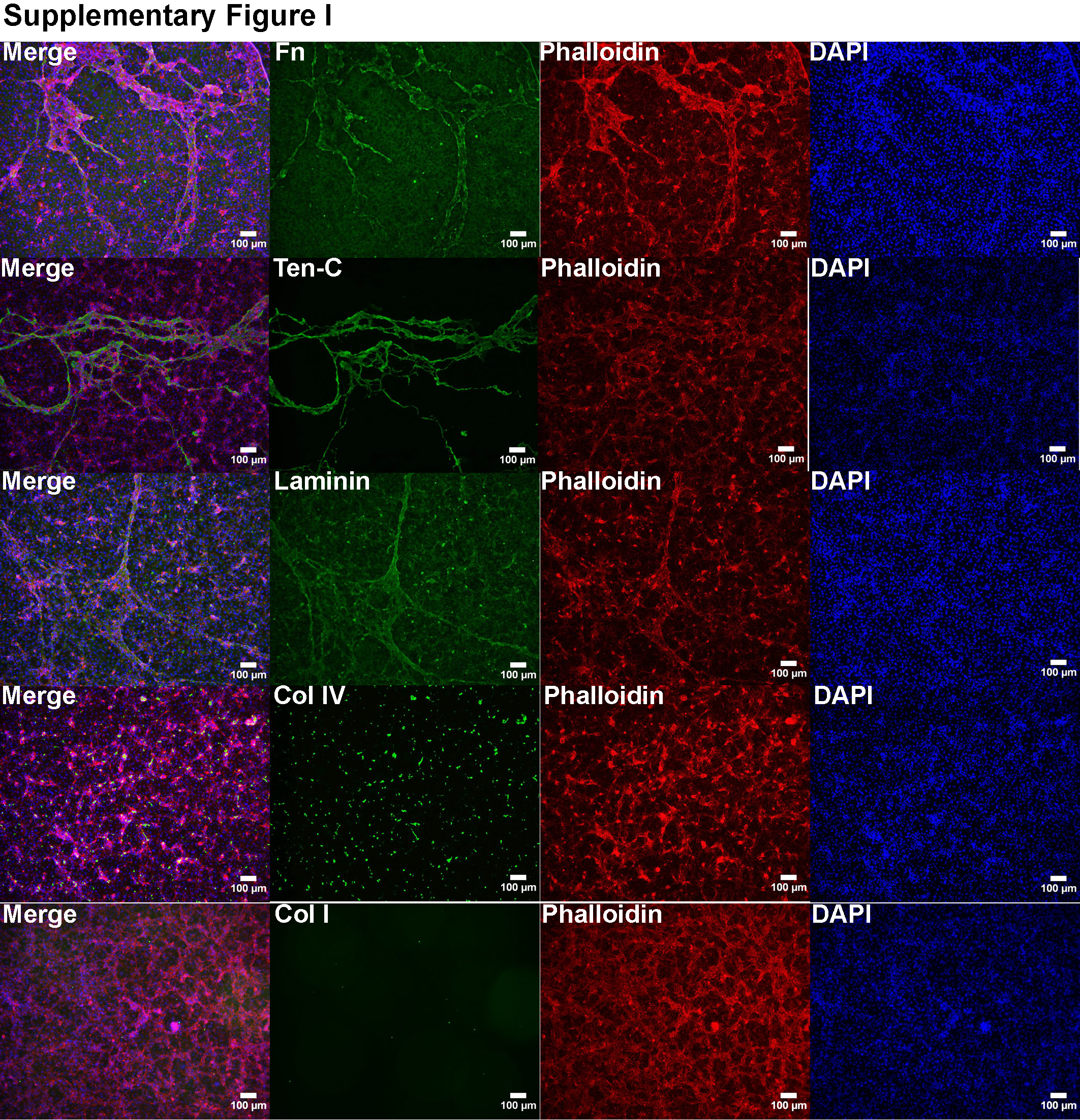

Supplement: S1 Fig — Low magnification immunofluorescence images of vascular structures depict the overall presentation of ECM proteins collagens I and IV, tenascin-C and fibronectin following vascular morphogenesis of ECs on de-cellularized co-culture ECM. (TIF) [file pone.0147600.s001.tif]

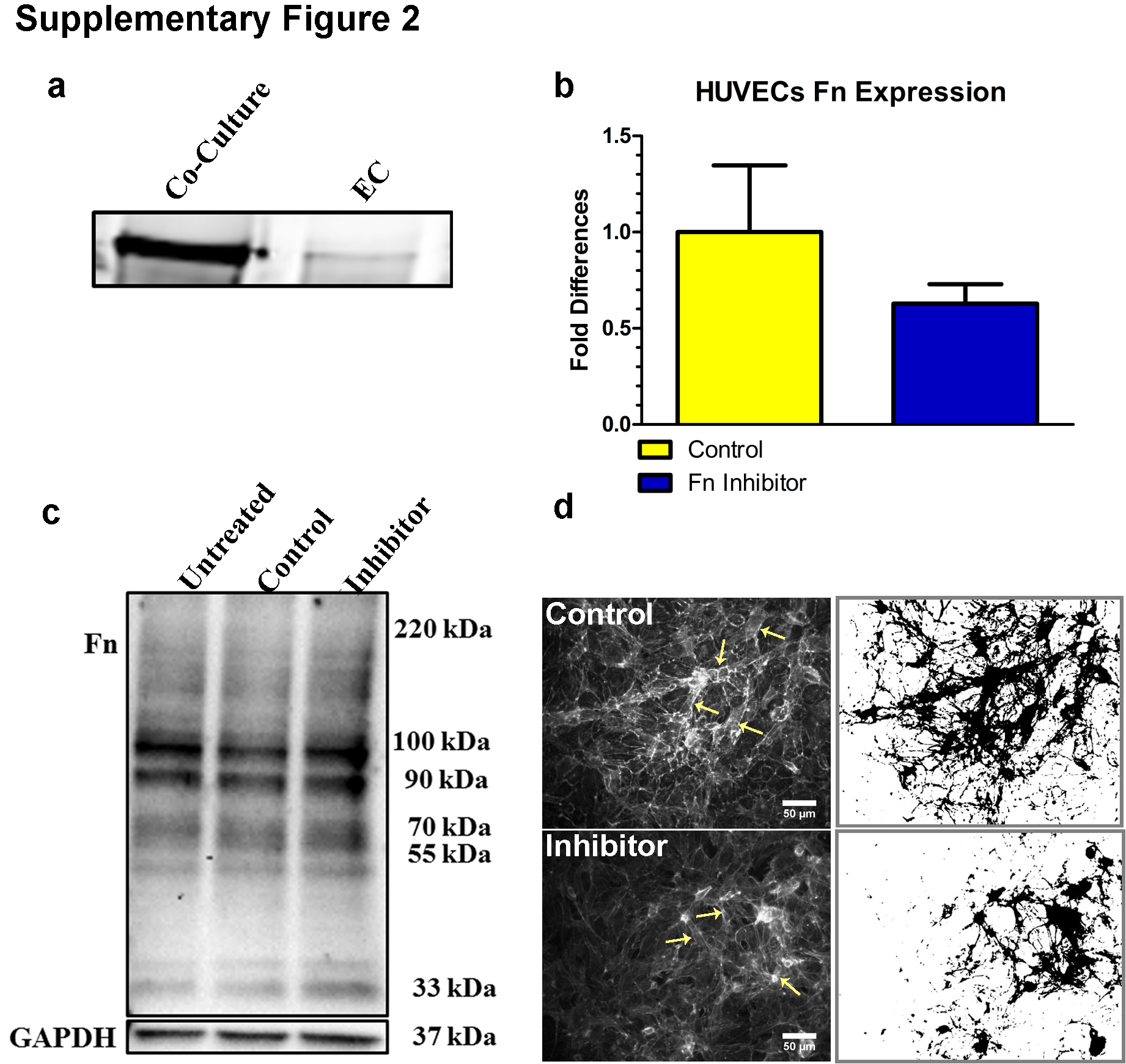

Supplement: S2 Fig — (A) Baseline fibronectin expression in co-culture and EC media. Results show fibronectin expression in the absence of cell produced fibronectin. (B) qRT-PCR and (C) western blot of fibronectin expression in ECs treated with pUR4B and control III-11C peptides at the time of seeding on de-cellularized co-culture ECM. *p≤0.05; **p≤0.01; ***p≤0.001. (D) Images of phalloidin-stained vascular structures before and after threshold using ImageJ. Thresholded images were used for analysis of the percent area occupied by CLS. Arrows indicate the presence of CLS in non-thresholded images. (TIF) [file pone.0147600.s002.tif]

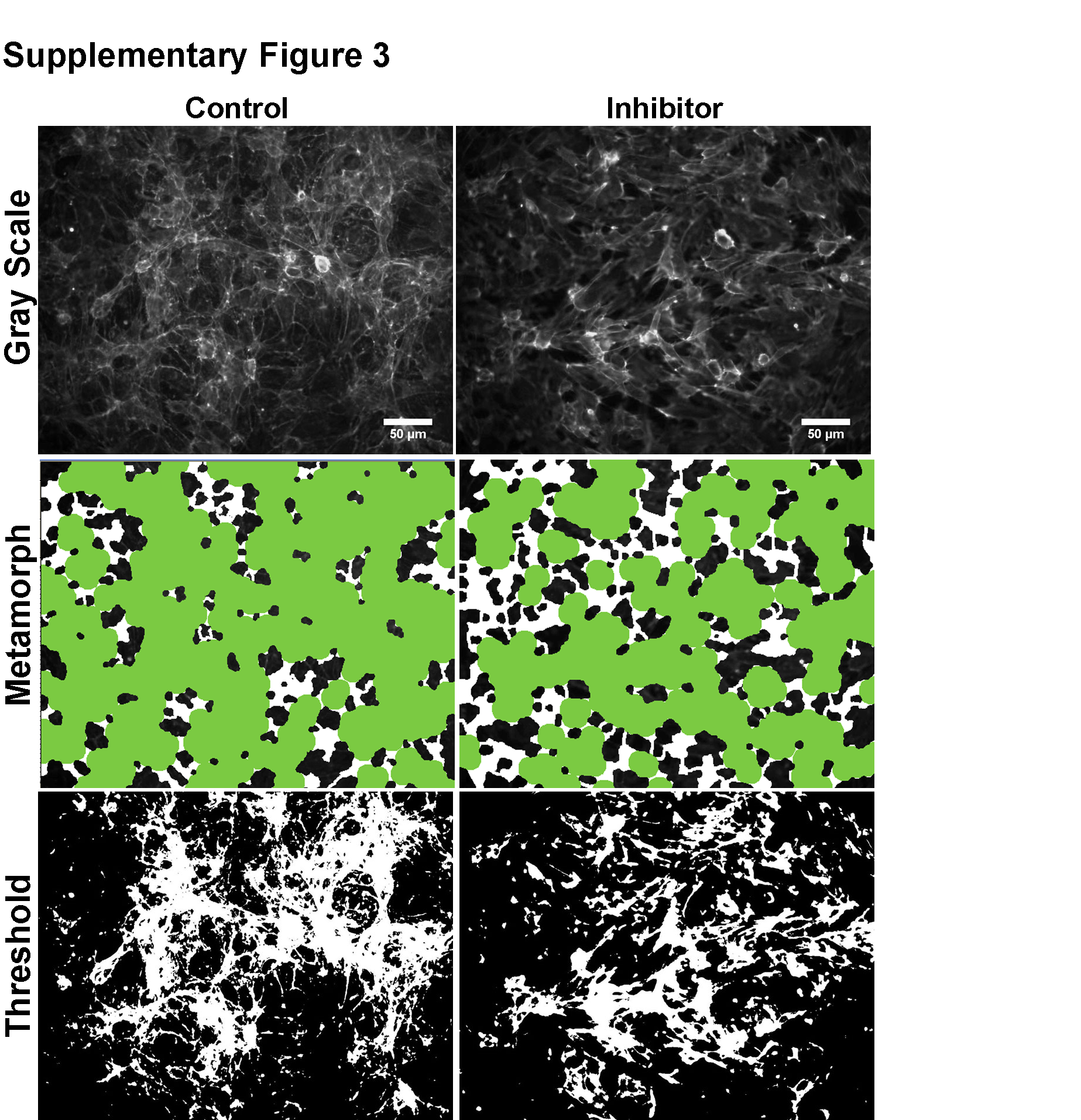

Supplement: S3 Fig — Images of CLS were generated using the angiogenesis tool in Metamorph. Images are from pUR4B (inhibitor) and control III-11C treated ECs cultured for 24 hours atop de-cellularized ECM matrices. The white regions are indicative of vascular structures while the green regions are indicative of nodes or vascular junctions. These nodes occupy a majority of the highlighted regions in both images. For comparison, the same images are shown before (top panel) and after thresholding (bottom panel), which was used to identify structures based on differences in pixel intensity. (TIF) [file pone.0147600.s003.tif]

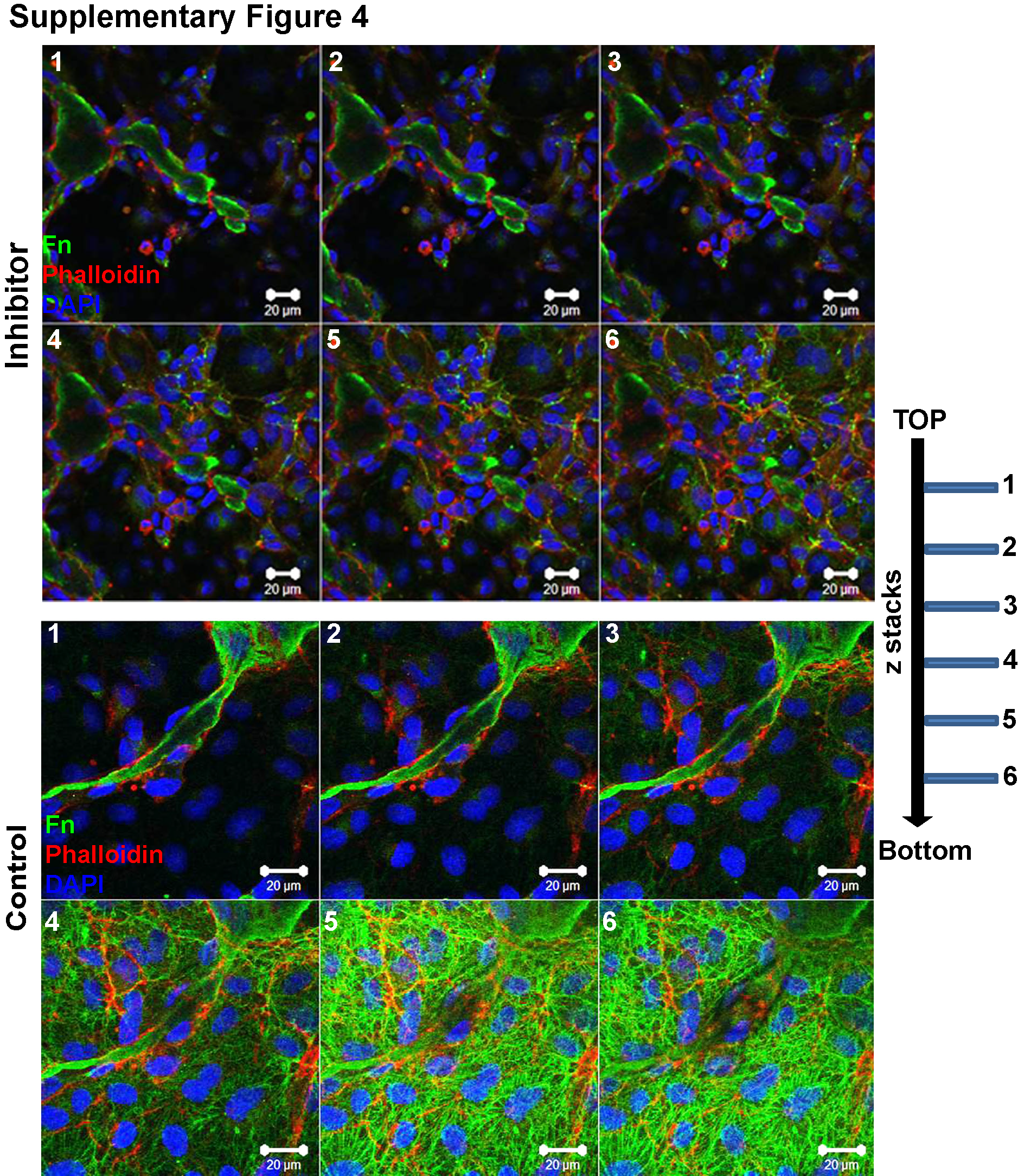

Supplement: S4 Fig — Confocal images of vascular structures treated with pUR4B and control III-11C peptides at the time of seeding on de-cellularized ECM. Images for pUR4B and control III-11C illustrate fibronectin expression in the first and last 3 sets of z stacks for vascular structures. Image 1 corresponds to the top of the well while image 6 corresponds to the bottom of the well. The intensity of fibronectin expression was greatest for vascular structures treated with control III-11C and lowest for vascular structures treated with the pUR4B inhibitor. Images were obtained using the same microscope settings. (TIF) [file pone.0147600.s004.tif]

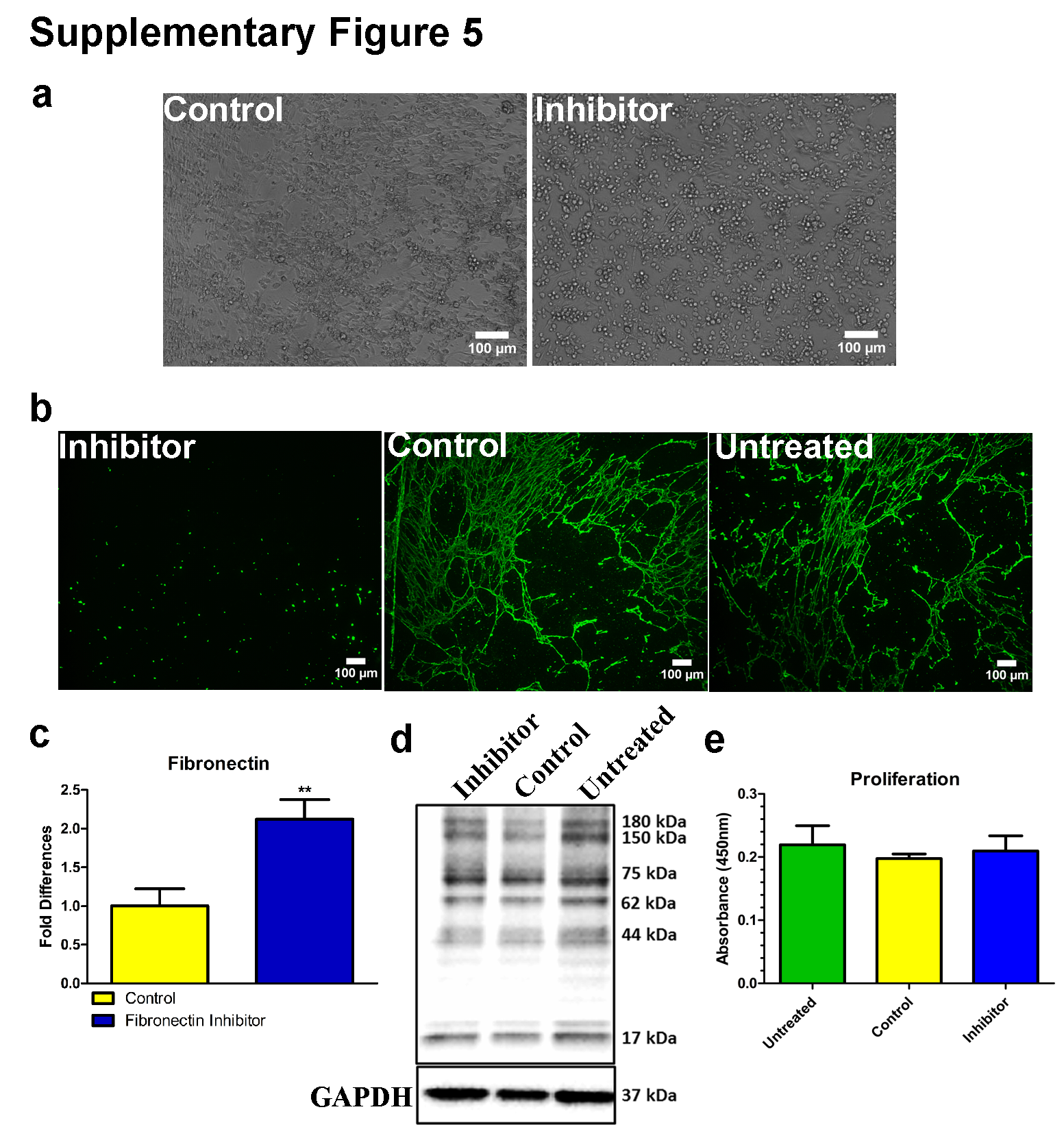

Supplement: S5 Fig — (A) Representative phase contrast images of pUR4B treated and untreated NuFF/MDA231 co-cultures prior to de-cellularization. (B) Representative immunofluorescence images of fibronectin in de-cellularized ECM from untreated, control III-11C treated and pUR4B treated co-cultures. (C) qRT-PCR and (D) western blot of fibronectin expression in control III-11C treated and pUR4B treated co-cultures. (E) Analysis of cell proliferation in untreated, control III-11C treated and pUR4B treated co-cultures. *p≤0.05; **p≤0.01; ***p≤0.001. (TIF) [file pone.0147600.s005.tif]

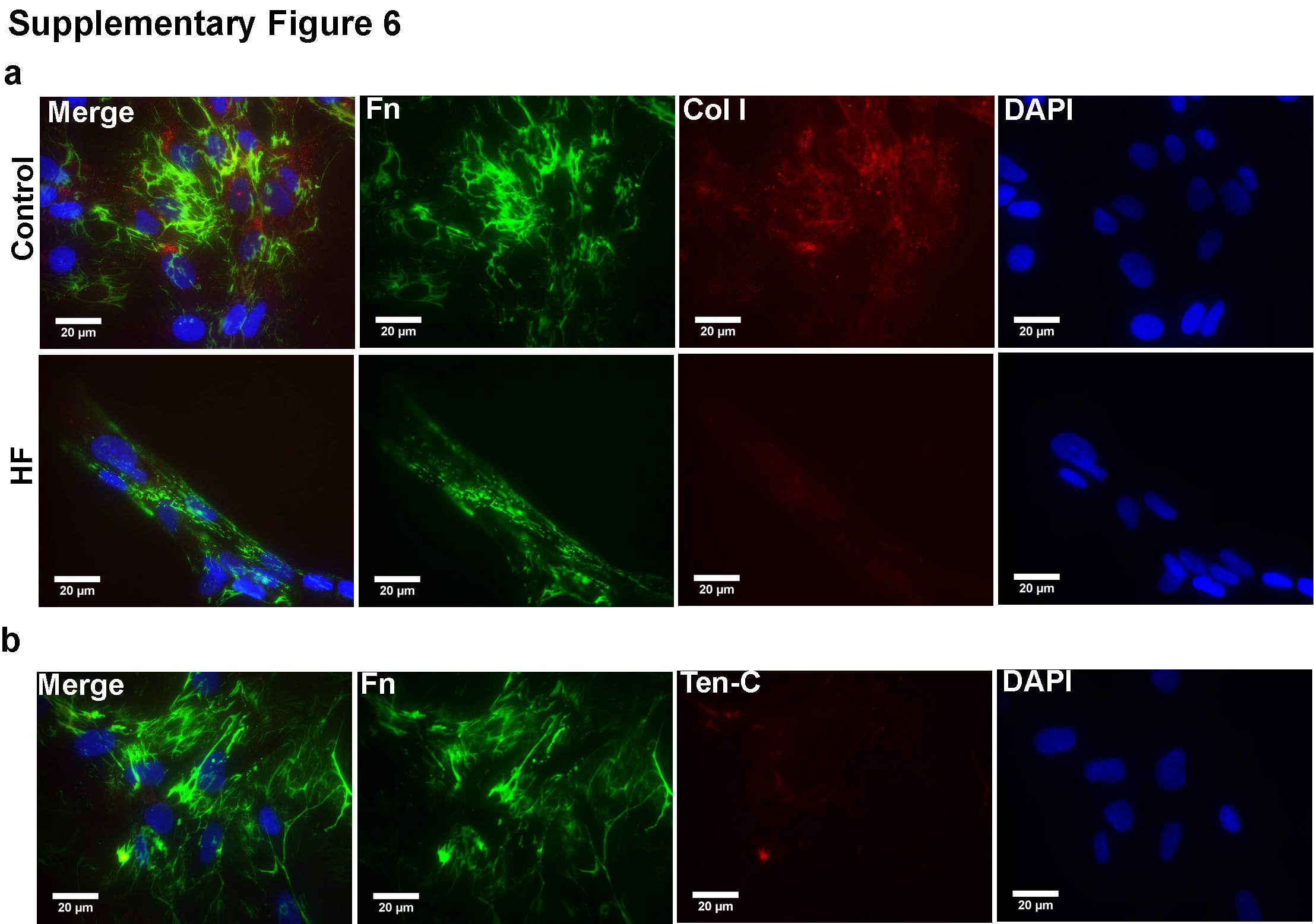

Supplement: S6 Fig — (A) Representative immunofluorescence images of NuFF cells treated with 100nM of halofuginone for 24 hours. Collagen I fibrils are observed with fibronectin fibrils in control NuFF cells, but are absent in halofuginone treated NuFF despite the presence of extracellular fibronectin fibrils. (B) Immunofluorescence images of control NuFF cells illustrate the absence of tenascin-C where fibronectin fibrils are observed. HF: Halofuginone. (TIF) [file pone.0147600.s006.tif]

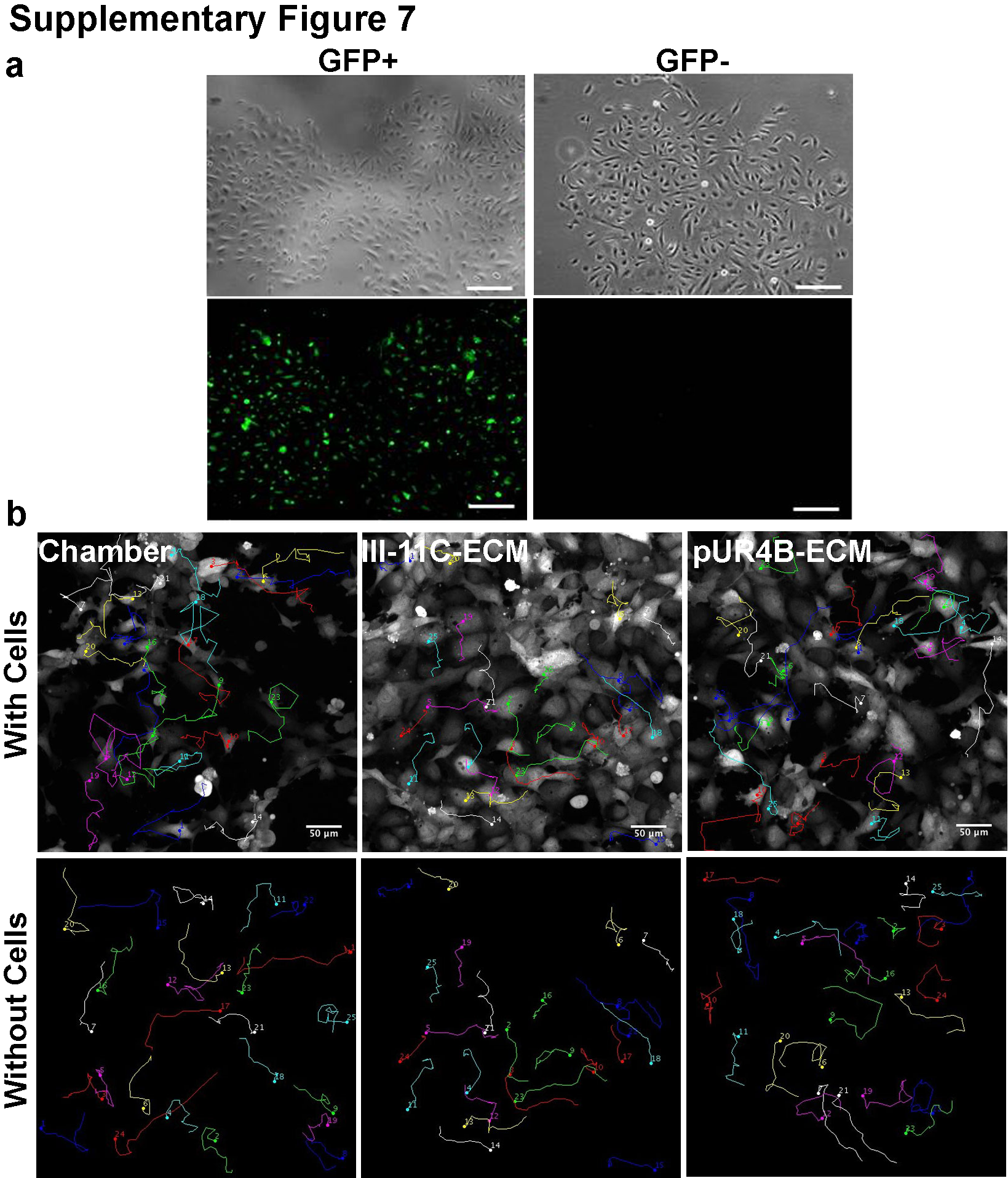

Supplement: S7 Fig — (A) ECs were transduced with lentiviral GFP and were sorted into GFP+ and GFP- subpopulations. Corresponding phase contract and fluorescence images were taken of both populations. These images depict the high fluorescence observed for the GFP+ subpopulation and the lack of fluorescence observed for the GFP+ subpopulation. Scale bars = 100μM. (B) The migration of the GFP+ ECs was monitored by tracking their positions every 15 minutes for a total of 5 hours. These images show the cells’ trajectories after the 5-hour time period. The top panel depicts trajectories with cells and the bottom panel depicts trajectories without the cells. The ECs are in grey scale while different colored lines represent the trajectories of random selected cells. The 5-hour trajectories were used to determine the total distance traveled by the ECs on each of the scaffolds. (TIF) [file pone.0147600.s007.tif]

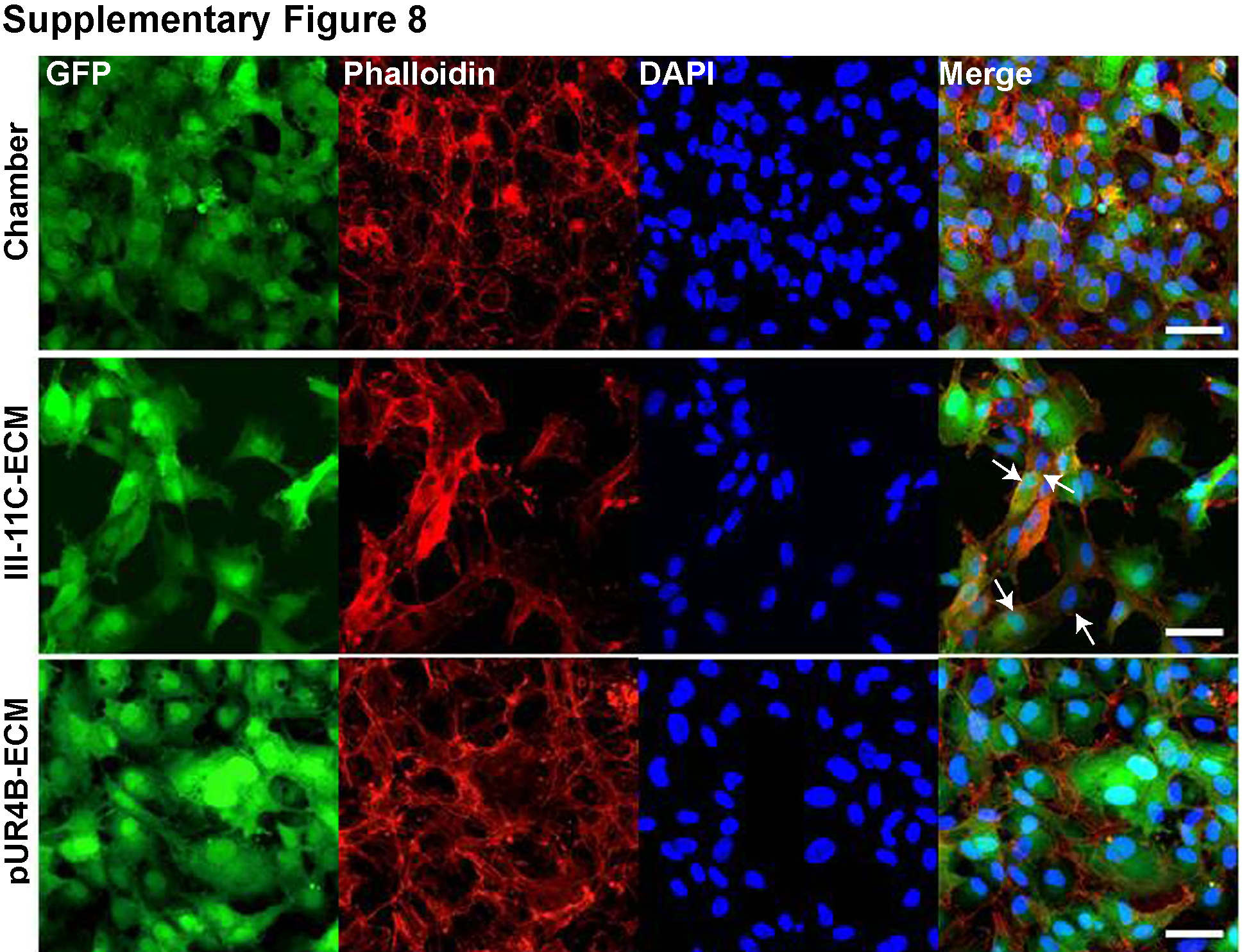

Supplement: S8 Fig — Confocal images were taken of GFP+ EC organization following growth on Chambers, III-11C-ECM and pUR4B-ECM. All images were acquired 12 hours post-seeding. Differences in vascular organization were evident from each of the tested conditions. ECs on Chambers and pUR4B-ECM have a sheet-like morphology with no evidence of nuclear alignment. ECs on III-11C-ECM exhibit vascular organization, evident by the presence of nuclear alignment into branched-like structures. These organized structures are indicated with white arrows. Scale bars = 100μM. (TIF) [file pone.0147600.s008.tif]
