# Supplementary material for: Fibronectin Deposition Participates in Extracellular Matrix Assembly and Vascular Morphogenesis
Source: PLoS One. 2016 Jan 26;11(1):e0147600. doi: 10.1371/journal.pone.0147600 (PMC4728102; doi:10.1371/journal.pone.0147600)
Supplement: S1 Table — (DOCX) [file pone.0147600.s009.docx]

**Supplementary Table 1: List of Antibodies**

| **Antibody** | **Concentration** | **Vendor** |
| --- | --- | --- |
| Fibronectin | 1:200 IF; 1:400 WB | Sigma |
| Tenasin-C | 1:100 IF | Santa Cruz |
| Collagen I | 1:350 IF | Abcam |
| Collagen IV | 1:100 IF | Abcam |
| Laminin | 1:100 IF | Abcam |
| MT1-MMP | 1:1,000 WB | Abcam |
| GAPDH | 1:3,000 WB | Cell Signaling Technology |
| CD31 | 1:200 IF | Dako |
| Phalloidin | 1:200 IF | Molecular Probes |
| Alexa fluor FITC | 1:1,000 IF | Life Technologies |
| Cy3 | 1:100 IF | Sigma |
| FITC | 1:100 IF | Sigma |
| HRP anti-rabbit | 1:1,000 WB | Cell Signaling Technology |

IF: Immunofluorescence; WB: Western Blot
